# Thalamo-cortical inter-subject functional correlation during movie watching across the adult lifespan

**DOI:** 10.3389/fnins.2022.984571

**Published:** 2022-09-21

**Authors:** Jinpeng Niu, Zihao Zheng, Ziqi Wang, Longchun Xu, Qingmin Meng, Xiaotong Zhang, Liangfeng Kuang, Shigang Wang, Li Dong, Jianfeng Qiu, Qing Jiao, Weifang Cao

**Affiliations:** ^1^Department of Radiology, The Second Affiliated Hospital of Shandong First Medical University, Tai’an, China; ^2^Department of Radiology, Shandong First Medical University and Shandong Academy of Medical Science, Tai’an, China; ^3^Ministry of Education (MOE) Key Laboratory for Neuroinformation, School of Life Sciences and Technology, The Clinical Hospital of Chengdu Brain Science Institute, University of Electronic Science and Technology of China, Chengdu, China; ^4^Department of Interventional Radiology, Taian Central Hospital, Tai’an, China

**Keywords:** fMRI, inter-subject functional correlation, thalamo-cortical, movie, aging

## Abstract

An increasing number of studies have shown that the functional interactions between the thalamus and cerebral cortices play an important role in cognitive function and are influenced by age. Previous studies have revealed age-related changes in the thalamo-cortical system within individuals, while neglecting differences between individuals. Here, we characterized inter-subject functional correlation (ISFC) between the thalamus and several cortical brain networks in 500 healthy participants aged 18–87 years old from the Cambridge Centre for Aging and Neuroscience (Cam-CAN) cohort using movie-watching state fMRI data. General linear models (GLM) were performed to assess age-related changes in ISFC of thalamo-cortical networks and the relationship between ISFC and fluid intelligence. We found significant age-related decreases in ISFC between the posterior thalamus (e.g., ventral posterior nucleus and pulvinar) and the attentional network, sensorimotor network, and visual network (FDR correction with *p* < 0.05). Meanwhile, the ISFC between the thalamus (mainly the mediodorsal nucleus and ventral thalamic nuclei) and higher-order cortical networks, including the default mode network, salience network and control network, showed complex changes with age. Furthermore, the altered ISFC of thalamo-cortical networks was positively correlated with decreased fluid intelligence (FDR correction with *p* < 0.05). Overall, our results provide further evidence that alterations in the functional integrity of the thalamo-cortical system might play an important role in cognitive decline during aging.

## Introduction

The thalamus is considered as a relay station to send information from the sensory periphery to the cerebral cortex (Moustafa et al., 2017). Evidence from anatomical studies suggests that the thalamus is a complex structure with multiple subdivisions that project to extensive cortical regions (Behrens et al., 2003; Sherman, 2016). Using diffusion tensor imaging, Behrens et al. (2003) found that the mediodorsal (MD) and ventral anterior (VA) nuclei project to the prefrontal cortex, and parts of the inferior pulvinar project to the temporal lobe. However, the ventral lateral (VL) and ventral posterior (VP) nuclei are functionally connected to the motor and premotor cortex (Hale et al., 2015). The connections between the thalamus and cerebral cortex play a critical role in movement, perception, attention, and execution (Moustafa et al., 2017; Anton-Bolanos et al., 2018). More importantly, the thalamo-cortical circuit is established from early embryonic stages and changes with developmental and aging processes (Anton-Bolanos et al., 2018) and is associated with cognitive decline in aging (Fama and Sullivan, 2015). Accumulating studies have also demonstrated that cognitive behavioral changes in some neurological diseases, such as mild cognitive impairment (Wang et al., 2012; Kim et al., 2020), Alzheimer’s disease (Jagirdar and Chin, 2019) and multiple sclerosis (Schoonheim et al., 2021), are associated with alterations in the thalamo-cortical circuit. Hence, a better understanding of thalamo-cortical interactions is essential for understanding the neurophysiological mechanisms of aging and neurodegenerative diseases.

As an important non-invasive imaging technique, functional magnetic resonance imaging (fMRI) has been increasingly used in the research of brain function during aging in recent years. Converging studies have shown that age-related cognitive decline is associated with functional changes in extensive cortical regions (Yang et al., 2016; Cohen et al., 2019). Convergent evidence from resting state and task state fMRI studies consistently showed that distinct cortical regions were organized into several brain networks associated with specific sensory and cognitive functions, such as the visual network (VN), attention network, control network (CN), salience network (SN), and default mode network (DMN) (Finn et al., 2015; Cao et al., 2016). In view of the role of the thalamus in multiple cognitive functions, accumulating studies have concentrated on thalamo-cortical networks and the influence of thalamocortical interactions on cognitive function with aging (Hatta et al., 2020; Das et al., 2021). Using resting-state fMRI, Steiner et al. (2020) found that the functional connectivity (FC) between the thalamus and SN and DMN decreases from 5 to 25 years old (Steiner et al., 2020). Using resting-state fMRI data of infants, Alcauter et al. (2014) demonstrated the presence of FC between the thalamus and SN in neonates, whereas FC between the thalamus and DMN emerges at 1 year of age (Alcauter et al., 2014). Moreover, changes in the connections between the thalamus and SN and DMN during aging are associated with altered cognitive function, such as processing speed, working memory and selective attention (Alcauter et al., 2014; Steiner et al., 2020). These studies focused on thalamo-cortical connections detected by FC analysis within the individual brain. Recently, a novel method termed inter-subject functional correlation (ISFC) has been introduced to reveal the extent of the neural response by calculating interregional correlations between different brains exposed to the same stimulus (Simony et al., 2016; Nastase et al., 2019). ISFC analysis improves the signal-to-noise ratio (SNR) by separating stimulus-induced correlations, intrinsic neural dynamics and non-neuronal artifacts (Simony et al., 2016). The method does not require the model of the stimulus time course and can apply many natural stimulus paradigms, such as movie watching (Pajula et al., 2012). Using movie watching state fMRI data, Gao et al. (2020) found that inter-subject correlation analysis was able to reliably characterize individual differences in brain responses elicited by stimuli, and this capability was robust across movies with different contents. Using an ISFC analysis, Simony et al. (2016) revealed that the DMN is reconfigured to process information in narrative comprehension (Simony et al., 2016). Zhang and Liu (2021) revealed that the DMN and CN have flexible interactions with other networks while watching movies (Zhang and Liu, 2021). Consequently, ISFC analysis may be an effective way to investigate functional alternations of the thalamo-cortical networks across the adult lifespan.

Recently, naturalistic paradigms, such as watching movies, have received much attention due to their advantages in studying the human brain in more realistic and natural settings (Jaaskelainen et al., 2021). A previous study showed that highly reliable and time-locked responses within and between subjects are induced in many brain regions while watching a movie, such as in the prefrontal cortex, visual cortex and parietal cortex (Hasson et al., 2010). In this study, we aimed to investigate age-related changes in thalamo-cortical ISFC during movie watching and its relationship with cognitive function. Five hundred healthy participants aged 18–87 years with movie-watching state fMRI data were selected from the Cam-CAN dataset. First, the ISFC maps were calculated between the thalamus and cortical networks for all subjects using fMRI data. Second, we explored age-related alternations of ISFC in thalamo-cortical networks. Finally, we investigated the relationship between altered ISFC and fluid intelligence that declines with age.

## Materials and methods

### Participants

Six hundred forty-seven subjects with movie-watching state fMRI data were from part of the Cambridge Centre for Aging Neuroscience (Cam-CAN)^1^ dataset (Shafto et al., 2014; Taylor et al., 2017). For each participant, detailed demographic data statistics, a self-administered health and lifestyle questionnaire and Mini-Mental State Exam (MMSE) were performed. The inclusion criteria included the following: score on the MMSE of 25 or above; good hearing (enable to hear 35 dB in either ear); native English speakers or bilingual English-speakers from birth; without MRI contraindications and neurological disorders. Sixty-seven subjects with excessive head movement (translation > 2.5 mm, rotation > 2.5°) and eighty subjects with missing fluid intelligence test scores were excluded from the present study. Five hundred participants (18–87 years old, mean = 51.75, standard deviation = 17.24, 248 males, and 252 females) were included in the final study (see Supplementary Table 1). All participants had complete demographic data, imaging data and cognitive indicators (fluid intelligence scores). Moreover, all participants met the experimental requirements and provided written informed consent in this study. This study was approved by the local ethics committee, Cambridgeshire 2 Research Ethics Committee, and was conducted in accordance with the principles of the Declaration of Helsinki.

### Movie stimuli

As a naturalistic stimulus, movies have been widely used in human neuroimaging studies and have yielded significant advances in the understanding of cognitive functions (Kauppi et al., 2010; Jaaskelainen et al., 2021). During the movie watching state scan in the present study, participants were instructed to watch, listen and pay attention to a black-and-white television drama by Alfred Hitchcock called “Bang! You’re Dead.” This movie is about a young boy who plays with a half-loaded revolver as a toy gun at home and in public, which can orchestrate the responses of so many different brain regions across participants associated with a more realistic setting (Hasson et al., 2008, 2010). Because of time constraints for MRI scans, the full 30-min episode was condensed to 8 min while maintaining the plot (Shafto et al., 2014). No participants knew what the movie was about, nor had they seen it before.

### Fluid intelligence test

In the present study, fluid intelligence was used as a measure of cognitive function as it had broad positive correlations with other cognitive tests (Shafto et al., 2014). The Cattell Culture Fair Test was used to measure the fluid intelligence of participants. The test is a pen-and-paper test that contains four subtests with a series of non-verbal distractions: series completion (3 min), classification (4 min), matrices (3 min), and conditions (2.5 min). In each test, participants choose a response from multiple options and record it on paper. If the choice is correct, they score one point (46 points in total). Previous studies have shown that fluid intelligence declines with age (Gray et al., 2003; Chen et al., 2020). Pearson correlation analysis of age and fluid intelligence was also conducted in the present study.

### Image acquisition

All MR measures were conducted on a 3T Siemens TIM Trio System with a 32-channel head coil (Shafto et al., 2014; Dachena et al., 2019). A high resolution (1 mm × 1 mm × 1 mm) 3D T1-weighted structural image was acquired using a Magnetization Prepared Rapid Gradient Echo (MPRAGE) sequence (repetition time (TR) = 2,250 ms; echo time (TE) = 2.99 ms; inversion time (TI) = 900 ms; field of view (FOV) = 256 mm × 240 mm × 192 mm; flip angle = 9 degrees; accelerated factor = 2) with an acquisition time of 4 min and 32 s. During movie watching, functional images were acquired using a multi-echo, T2*-weighted EPI sequence with the following parameters: TR = 2,470 ms; 5 echoes (TE = 9.4, 21.2, 33, 45, 57 ms); FOV = 192 mm × 192 mm; voxel size = 3 mm × 3 mm × 4.44 mm; flip angle = 78 degrees; number of axial slices = 32 (acquired in descending order); slice thickness = 3.7 mm (with 20% interslice gap); acquisition time = 8 min and 13 s.

### Imaging analyses

#### Data preprocessing

For fMRI images, the preprocessing was conducted using Statistical Parametric Mapping (SPM12)^2^ from the Neuroscience Information Toolbox (NIT)^3^ (Dong et al., 2018). FMRI images were preprocessed as follows: (1) Removing the first five functional volumes to ensure magnetic field stabilization; (2) Slice-timing correction; (3) Realigning for head motion correction. Subjects whose head movement exceeded 2.5 mm (translation) or 2.5° (rotation) were excluded. Furthermore, the mean framewise displacement (mFD) of all participants in this study was calculated, and the threshold of mFD was chosen at 0.5 to discard the bad time points of images (Power et al., 2012; Gao et al., 2020); (4) Registering individual T1 images to functional images. The T1 images were segmented into gray matter (GM), white matter (WM), and cerebrospinal fluid (CSF). And brain volume was extracted for each subject based on individual T1 image; (5) Normalizing the functional images to the Montreal Neurological Institute (MNI) template (3 × 3 × 3 mm^3^) using parameters from individual T1 segmentation; (6) Spatial smoothing of the remaining functional images with an 8 mm full width at half maximum (FWHM) Gaussian kernel. Pajula and Tohka (2014) found that the FWHM of spatial smoothing larger than twice the voxel size was a good selection for the spatial registration accuracy and decent signal noise ratio (SNR) in inter-subject analysis; (7) Detrending and bandpass filtering (0.01∼0.08 Hz) were applied to reduce low-frequency drift and high-frequency noise; (8) Nuisance regression, including 12 head motion parameters (six head-motion parameters and their derivatives), WM signal and CSF signal.

#### Inter-subject functional correlation analysis

A 246-region of interest (ROIs) brain atlas containing anatomical and functional connection information was selected in this study (Fan et al., 2016). The time courses of each ROI were obtained by averaging the time courses of all voxels within the ROI. Seventeen cortical networks were defined based on a network atlas from Yeo, including the visual peripheral network (VPN), visual central network (VCN), sensorimotor network A (SMN_A), sensorimotor network B (SMN_B), dorsal attention network A (DAN_A), dorsal attention network B (DAN_B), ventral attention network (VAN), salience network (SN), limbic network A (Limbic_A), limbic network B (Limbic_B), control network A (CN_A), control network B (CN_B), control network C (CN_C), default mode network A (DMN_A), default mode network B (DMN_B), default mode network C (DMN_C), and default mode network D (DMN_D) (Yeo et al., 2011). The brain regions contained in each network are described in Supplementary Table 2. In addition, seven networks from Yeo were also used to explore the relationships between ISFCs of thalamo-cortical networks and age and fluid intelligence, including the visual network (VN), sensorimotor network (SMN), dorsal attention network (DAN), VAN, limbic network (Limbic), frontoparietal network (FPN), and default mode network (DMN) (Yeo et al., 2011). The mask of the thalamus was defined based on the Anatomical Automatic Labeling (AAL) atlas (Tzourio-Mazoyer et al., 2002). With downsampling to the standard MNI space (Tzourio-Mazoyer et al., 2002), 620 voxels were included in the entire thalamus.

To explore the relationship between the ISFC and age, as well as fluid intelligence, an ISFC matrix between the thalamus and cortical networks was constructed for each subject. First, according to the influence of sample size on inter-subject analysis and the board age span (Pajula and Tohka, 2016; Geerligs et al., 2018), all subjects were divided into seven groups with an age range of 10 years (i.e., 18–27 years old, 28–37 years old. 78–87 years old, see Table 1). Second, Pearson correlation was calculated between the mean time course of each brain network of the subject and time courses of thalamic voxels of the other subjects in the group, resulting in n-1 ISFC matrices (17 × 620) of all subjects-pairs in each group with n subjects. Finally, to obtain the final ISFC matrix of the subject, ISFC values of all subjects-pairs were averaged. Fisher r-z transformation was applied to each correlation coefficient (Berry and Mielke, 2000; Simony et al., 2016). The mean ISFC matrix of each group is shown in Supplementary Figure 1. A one-sample *t*-test was performed to detect significant thalamo-cortical network ISFCs in all subjects, and the resulting ISFCs (survived after false discovery rate correction with *p* < 0.05, see Supplementary Figure 2) were used for further regression analyses. In addition to grouping with an age range of 10 years, we also grouped all participants by different age ranges and performed ISFC analysis, respectively, including 5, 15, 20, 25, and 30 years.

**TABLE 1:** Demographic data and cognitive measures of participants in each group.

Group	1	2	3	4	5	6	7
Age range (year)	18–27	28–37	38–47	48–57	58–67	68–77	78–87
Number	33	98	94	88	72	66	49
Male/female	13/20	48/50	47/47	44/44	36/36	37/29	23/26
Education (year)	21.12 ± 2.81	22.27 ± 3.17	21.52 ± 3.40	20.55 ± 3.18	19.86 ± 3.88	19.77 ± 4.74	19.24 ± 3.90
MMSE	29.36 ± 0.99	29.53 ± 0.86	29.07 ± 1.14	29.20 ± 1.06	29.04 ± 1.12	28.69 ± 1.26	28.29 ± 1.49
Fluid intelligence	38.03 ± 3.19	37.28 ± 4.25	35.15 ± 4.27	33.64 ± 4.33	30.89 ± 5.49	27.39 ± 5.77	24.31 ± 5.61

The education is defined as the age of completing full-time education. Education, MMSE and fluid intelligence are expressed as Mean ± Standard deviation. MMSE, Mini-Mental State Exam.

#### General linear model analysis

**FIGURE 1:**
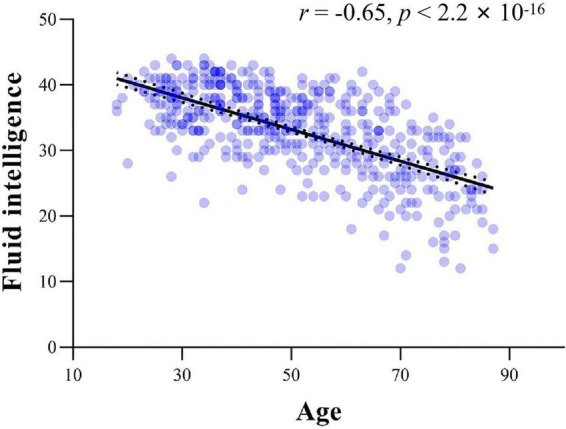
The scatter plot between age and fluid intelligence.

To investigate the changes in ISFCs between the thalamus and cortical networks across the adult lifespan, a general linear model (GLM) was used while adding gender, education, mFD and brain volume as covariates (Equation 1) (Poline and Brett, 2012). Meanwhile, the quadratic relationship between the ISFC and age was also explored in the present study (Supplementary material 2). As the age and fluid intelligence scores were strongly correlated (see Figure 1), adding the fluid intelligence scores in Equation 1 would lead to a collinearity problem (Kievit et al., 2014). Thus, an additional GLM analysis was performed to determine the relationship between ISFCs and fluid intelligence with the same covariates (Equation 2). To test the significance of the regression coefficients (β_1_, β_2_, β′_1_), the *T*-values were calculated for each regression coefficient to measure the relationships between ISFC and age, as well as fluid intelligence. The resulting *p*-values of thalamo-cortical network ISFCs were corrected with false discovery rate (FDR) correction based on multiple comparisons (*p* < 0.05) (Benjamini and Yosef, 1995).


(1) $$\begin{array}{c} I\,S\,F\,C=\beta_{0}+\beta_{1}\cdot a\,g\,e+\beta_{2}\cdot a\,g\,e^{2}+\beta_{3}\cdot s\,e\,x+\\ \beta_{4}\cdot e\,d\,u\,c\,a\,t\,i\,o\,n+\beta_{5}\cdot m\,F\,D+\beta_{6}\cdot v\,o\,l\,u\,m\,e \end{array}$$

(2) $$\begin{array}{c} I\,S\,F\,C=\beta_{0}^{\prime }+\beta_{1}^{\prime }\cdot b\,e\,h\,a\,v\,i\,o\,r+\beta_{2}^{\prime }\cdot s\,e\,x+\beta_{3}^{\prime }\cdot e\,d\,u\,c\,a\,t\,i\,o\,n\\ +\beta_{4}^{\prime }\cdot m\,F\,D+\beta_{5}^{\prime }\cdot v\,o\,l\,u\,m\,e\;\;\;\;\; \end{array}$$

#### Split-half validation analysis

To verify the reliability of the results, half of the participants were randomly selected. These subjects were divided into seven groups with an age range of 10 years (i.e., 18–27 years old, 28–37 years old, 78–87 years old, see Supplementary Table 3). First, the ISFC matrix between the thalamus and cortical networks was calculated for each subject within each group. Second, the GLM (Equation 1) was used to explore the changes in ISFCs between the thalamus and cortical networks across the adult lifespan in validation set, with gender, education, mFD and brain volume as covariates. Finally, the relationship between ISFCs and fluid intelligence was investigated using an GLM (Equation 2) with the same covariates.

## Results

### Changes in inter-subject functional correlation with age between the thalamus and cortical networks

**FIGURE 2:**
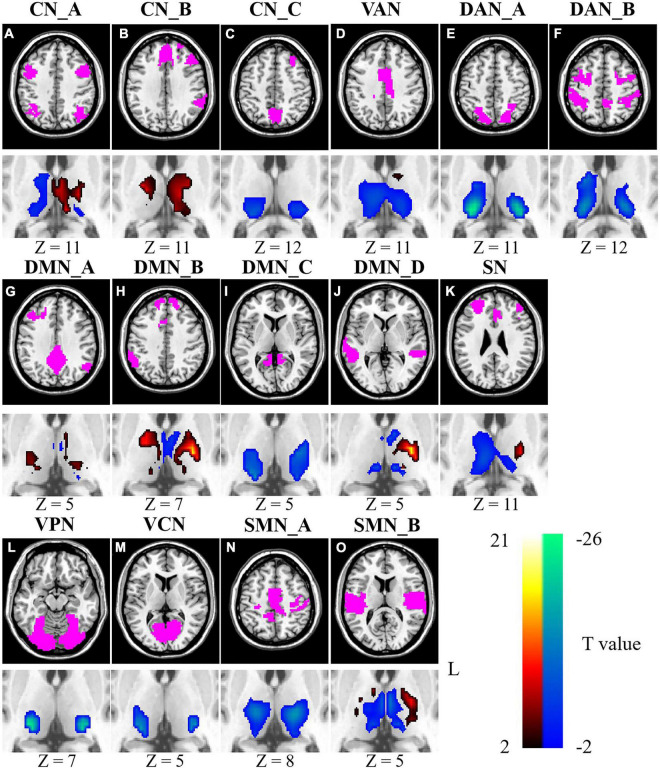
Age-related changes of ISFCs between the thalamus and cortical networks **(A–O)**. Color bar signifies the T statistics (warm color, ISFCs increased significantly as age increased; cool color, ISFCs decreased significantly as age increased; FDR-corrected). L represents the left hemisphere. A minimum cluster size was 23 adjacent voxels in the thalamus. CN_A, control network A; CN_B, control network B; CN_C, control network C; VAN, ventral attention network; DAN_A, dorsal attention network A; DAN_B, dorsal attention network B; DMN_A, default mode network A; DMN_B, default mode network B; DMN_C, default mode network C; DMN_D, default mode network D; SN, salience network; VPN, visual peripheral network; VCN, visual central network; SMN_A, sensorimotor network A; SMN_B, sensorimotor network B.

As shown in Figure 2, the ISFCs between the thalamus and cortical networks demonstrated age-related changes during movie watching. For CN, age-related decreases in ISFC were found in the bilateral pulvinar, right ventral lateral (VL) nucleus and ventral posterior (VP) nucleus (*p* < 0.0326). Age-related increases in ISFC were found between the left VL nucleus, VP nucleus, MD nucleus and CN_A and CN_B (*p* < 0.0222) (Figures 2A–C). Age-related decreases in ISFC were found between DMN_C and the VL nucleus, VP nucleus, and pulvinar (*p* < 0.0089). Moreover, the ISFCs between the MD nucleus and DMN_A, DMN_B and DMN_D decreased with age (*p* < 0.0361). Age-related increases in ISFC were found between DMN_B and part of the bilateral VA nucleus and VL nucleus (*p* < 0.0358). For DMN_D, age-related increases in ISFC were only found in part of the left VL nucleus (*p* < 0.0272) (Figures 2G–J). For SN, age-related decreases in ISFC were found in the MD nucleus, right VL nucleus and part of the pulvinar (*p* < 0.0365), while age-related increases were found in the left VL nucleus and part of the anterior thalamus (*p* < 0.0365) (Figure 2K). For VAN, age-related decreases in ISFCs were found mainly in the MD nucleus, VL nucleus, and pulvinar (*p* < 0.0365) (Figure 2D). For DAN, age-related decreases in ISFC were found in the lateral and posterior parts of the thalamus, mainly the pulvinar, VL nucleus and VP nucleus (*p* < 0.0361) (Figures 2E,F). For ISFC between the VN and thalamus, age-related decreases were observed in lateral and posterior subthalamic nuclei, including the VP nucleus and the pulvinar (*p* < 0.0026) (Figures 2L,M). Moreover, the ISFCs between SMN_A and the VL nucleus and VP nucleus were negatively correlated with age (*p* < 0.0326), and a positive correlation was found between SMN_B and the left VL nucleus and VP nucleus (*p* < 0.0255) (Figures 2N,O).

The ISFCs between the thalamus and seven cortical networks also showed age-related changes during movie watching. For FPN, age-related decreased ISFC was found in the right VP nucleus (*p* < 0.0269), while increased ISFC was found in the left VL nucleus and MD nucleus (*p* < 0.0349) (Supplementary Figure 6A). For DMN, age-related increases in ISFC were found in part of the VA nucleus and VL nucleus (*p* < 0.0321) (Supplementary Figure 6B). The ISFC between the VAN and MD nucleus and VL nucleus decreased with age (*p* < 0.0360) (Supplementary Figure 6C). For DAN, age-related decreases in ISFC were found in the lateral and posterior parts of the thalamus (*p* < 0.0271) (Supplementary Figure 6D). For VN, age-related decreases in ISFC were found in the VP nucleus and the pulvinar (*p* < 0.001) (Supplementary Figure 6E). For ISFC between the SMN and the thalamus, age-related decreases were mainly located in the MD nucleus (*p* < 0.0355) (Supplementary Figure 6F).

### Relationships between fluid intelligence and inter-subject functional correlations

**FIGURE 3:**
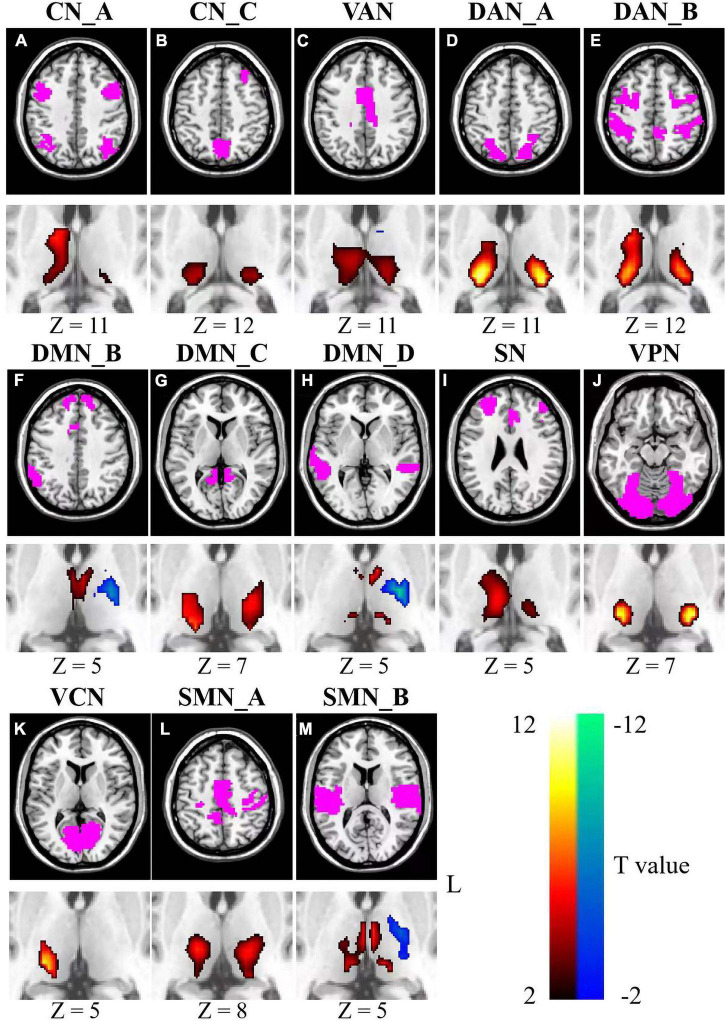
Relationships between fluid intelligence and ISFCs between the thalamus and cortical networks **(A–M)**. Color bar signifies the T statistics (warm color, ISFCs increased significantly as fluid intelligence scores increased; cool color, ISFCs decreased significantly as fluid intelligence scores increased; FDR-corrected). L represents the left hemisphere. A minimum cluster size was 23 adjacent voxels in the thalamus. CN_A, control network A; CN_C, control network C; VAN, ventral attention network; DAN_A, dorsal attention network A; DAN_B, dorsal attention network B; DMN_B, default mode network B; DMN_C, default mode network C; DMN_D, default mode network D; SN, salience network; VPN, visual peripheral network; VCN, visual central network; SMN_A, sensorimotor network A; SMN_B, sensorimotor network B.

A significant negative relationship between age and fluid intelligence scores was found across all participants (*r* = −0.65, *p* < 2.2 × 10^–16^, see Figure 1). Analyses revealed significant associations between fluid intelligence and ISFCs between the thalamus and cortical networks (Figure 3). For CN, positive associations between fluid intelligence and ISFCs were found in the bilateral pulvinar, right VL nucleus and VP nucleus (*p* < 0.0256), while negative associations for CN_A were found in part of the MD nucleus (*p* < 0.0240) (Figures 3A,B). For DMN_C, positive associations between fluid intelligence and ISFCs were found in the VL nucleus, VP nucleus and pulvinar (*p* < 0.0254) (Figure 3G). Moreover, significant positive relationships between fluid intelligence and ISFCs for DMN_B and DMN_D were found in the MD nucleus and VA nucleus (*p* < 0.0248), while negative relationships were found in part of the left VL nucleus (*p* < 0.0259) (Figures 3F,H). Significant positive relationships between fluid intelligence and ISFCs for the DAN, VAN and SN were found in the VL nucleus, VP nucleus and part of the pulvinar (*p* < 0.0257) (Figures 3C–E,I). For VN, significant positive associations between fluid intelligence and ISFCs were found in the VP nucleus and pulvinar (*p* < 0.0249) (Figures 3J,K). Moreover, we found positive correlations between fluid intelligence and ISFCs for SMN_A in the VL nucleus and VP nucleus (*p* < 0.0248), while negative correlations for SMN_B were found in the left VL nucleus and VP nucleus (*p* < 0.0249) (Figures 3L,M).

The ISFC between the thalamus and seven cortical networks also showed significant correlations with fluid intelligence. The ISFC between the DMN and left VL nucleus showed a negative relationship with fluid intelligence (*p* < 0.0246), but no significant relationship was found between the thalamus and the FPN (Supplementary Figures 7A,B). Significant positive relationships between fluid intelligence and ISFCs for the DAN and VAN were found in the VL nucleus, VP nucleus and part of the pulvinar (*p* < 0.0243) (Supplementary Figures 7C,D). For VN, positive associations between fluid intelligence and ISFCs were found in the VP nucleus and the pulvinar (*p* < 0.0249) (Supplementary Figure 7E). The ISFC between the SMN and MD nucleus showed a positive correlation with fluid intelligence (*p* < 0.0235) (Supplementary Figure 7F).

### Reproducibility of age-related changes in inter-subject functional correlation between the thalamus and cortical networks and its relationship with fluid intelligence

We randomly selected half of the participants and performed the ISFC analysis and GLM analysis. We found similar age-related changes in the ISFC of the thalamo-cortical networks (Supplementary Figure 8). For CN, age-related decreases in ISFC were found in the bilateral pulvinar, right ventral lateral (VL) nucleus and ventral posterior (VP) nucleus (*p* < 0.0281). Age-related increases in ISFC were found between the MD nucleus and CN_A (*p* < 0.0289) (Supplementary Figures 8A–C). Age-related decreases in ISFC were found between DMN_C and the VL nucleus, VP nucleus, and pulvinar (*p* < 0.0274). Moreover, the ISFCs between the MD nucleus and DMN_B and DMN_D decreased with age (*p* < 0.0281). Age-related increases in ISFC were found between DMN_B, DMN_D and part of the left VA nucleus and VL nucleus (*p* < 0.0287) (Supplementary Figures 8D–F). For SN, age-related decreases in ISFC were found in the right VL nucleus and part of the pulvinar (*p* < 0.0287) (Supplementary Figure 8G). The ISFCs between the MD nucleus and Limbic_A decreased with age (*p* < 0.0274) (Supplementary Figure 8H). For VAN, age-related decreases in ISFCs were found mainly in the MD nucleus, VL nucleus, and pulvinar (*p* < 0.0290) (Supplementary Figure 8I). For DAN, age-related decreases in ISFC were found in the lateral and posterior parts of the thalamus, mainly the pulvinar, VL nucleus and VP nucleus (*p* < 0.0256) (Supplementary Figures 8J,K). For ISFC between the VN and thalamus, age-related decreases were observed in the VP nucleus and the pulvinar (*p* < 0.0170) (Supplementary Figures 8L,M). Moreover, the ISFCs between SMN_A, SMN_B and the VL nucleus and MD nucleus were negatively correlated with age (*p* < 0.0287), and a positive correlation was found between SMN_B and the left VL nucleus (*p* < 0.0251) (Supplementary Figures 8N,O).

Meanwhile, similar relationships between the ISFC of thalamo-cortical networks and fluid intelligence were found in the split-half cross validation (Supplementary Figure 9). For CN, positive associations between fluid intelligence and ISFCs were found in the bilateral pulvinar, right VL nucleus and VA nucleus (*p* < 0.0175) (Supplementary Figures 9A,B). For DMN_C, positive associations between fluid intelligence and ISFCs were found in the VL nucleus, VP nucleus and pulvinar (*p* < 0.0164) (Supplementary Figure 9D). Moreover, significant positive relationships between fluid intelligence and ISFCs for DMN_B and DMN_D were found in the MD nucleus and VA nucleus (*p* < 0.0180) (Supplementary Figures 9C,E). For SN, positive associations between fluid intelligence and ISFCs were found in the right VA nucleus (*p* < 0.0179) (Supplementary Figure 9F). Significant positive relationships between fluid intelligence and ISFCs for the DAN were found in the VL nucleus, VP nucleus and part of the pulvinar (*p* < 0.0173) (Supplementary Figures 9G,H). For VN, significant positive associations between fluid intelligence and ISFCs were found in the VP nucleus and pulvinar (*p* < 0.0170) (Supplementary Figures 9I,J). Moreover, we found positive correlations between fluid intelligence and ISFCs for SMN_B in the MD nucleus (*p* < 0.0167) (Supplementary Figure 9K).

## Discussion

In the present study, we investigated changes in thalamo-cortical functional networks across the adult lifespan using ISFC analysis of fMRI under naturalistic stimuli. We found that the ISFC between the thalamus (located in the MD nucleus and ventral thalamic nuclei) and higher-order cognitive functional networks showed complex patterns of changes with age, including the SN, CN, and DMN. The ISFC between the VP nucleus and pulvinar of the thalamus and the VN, DAN, VAN, and SMN decreased with age. Furthermore, the alterations in ISFC of the thalamo-cortical networks were associated with age-related decline in fluid intelligence.

We found age-related decreases in ISFC between the SN and right ventral and posterior thalamus and increases in ISFC between the SN and left lateral thalamus while watching movies. The SN, mainly consisting of the anterior insula and anterior cingulate cortex (ACC), is implicated to be important in regulating cognitive resources to complete a cognitive process and is affected by aging (Chand et al., 2017). A 4-year longitudinal study of older adults showed decreased FC within the SN, suggesting that lower FC in the SN might indicate altered attention control in older adults (Oschmann and Gawryluk, 2020). Moreover, Yuan et al. (2016) found that the SN connects to the MD nucleus, pulvinar and a small portion of the lateral posterior nucleus (Yuan et al., 2016). Steiner et al. (2020) found age-related decreases in functional connections between the SN and the ventral thalamus, and decreased FC between the SN and right VA and VL nuclei is associated with decreased processing speed (Steiner et al., 2020). Using movie-watching state fMRI data in the present study, we found that reduced ISFC between the SN and right ventral and posterior thalamus was significantly associated with reduced fluid intelligence. Using resting-state fMRI data to investigate age-related alternations in FC between brain networks, Zhai and Li (2019) found that the FC between the SN and the DMN and subcortical network increases with age (Zhai and Li, 2019). Increased FC both within and between functional networks in older adults suggests a positive form of cognitive plasticity, which may reflect compensatory cognitive decline (Hakun et al., 2015; Zonneveld et al., 2019). Hence, age-related increases in ISFC between the SN and left lateral thalamus might suggest the upregulation of cognitive control while watching movies. Using resting state fMRI, Vij et al. (2018) found that the spatial extent of the SN exhibited negative quadratic trajectories across the lifespan, and the FC between the SN and SMN, VN also showed a negative quadratic relationship with age. In the present study, we found that the ISFC between the SN and right ventral and posterior thalamus showed a negative quadratic relationship with age.

We found age-related decreases in ISFC between the pulvinar and CN_C, which mainly contains the posterior parietal cortex (PPC). The ISFC between the medial and ventral thalamus and CN_A and CN_B showed a negative quadratic relationship with age, while increased ISFC was found between the anterior and medial thalamus and CN_A and CN_B, which consist of the lateral prefrontal cortex and the PPC. The lateral prefrontal cortex extensively connects to subcortical and posterior cortical brain regions for implementing cognitive processes and contributing to fluid intelligence, and the PPC is involved in multiple cognitive processes, including attention control (Cole et al., 2015; Whitlock, 2017). Campbell et al. (2015) demonstrated that the age-related decline in synchronization of the CN is more pronounced in unattractive parts of the movie, suggesting that older adults are more prone to cognitive or attentional control lapses (Campbell et al., 2015). During the working memory task, Charroud et al. (2016) found that increased FC within the CN is associated with better performance in older adults (Charroud et al., 2016). Evidence from diffusion tensor imaging studies showed that the PPC projects to the pulvinar, and the dorsolateral prefrontal cortex (DLPFC) and ventrolateral prefrontal cortex (VLPFC) connect with the MD nucleus (Behrens et al., 2003; Jang and Yeo, 2014). Using resting-state fMRI, Yuan et al. (2016) found that the CN is functionally connected with the pulvinar, MD and VL nuclei (Yuan et al., 2016). A recent functional imaging study demonstrated that the thalamus is a key hub to balance the functional activity of the SN, CN, and DMN with aging, and thalamo-cortical interactions are important for maintaining cognitive functions in older adults (Das et al., 2021). In the present study, we found that age-related decreases in ISFC between the pulvinar and the CN_C while watching movies were associated with decreased fluid intelligence, and increased ISFC between the medial thalamus and the CN_A was also associated with decreased fluid intelligence. We also found age-related declines in fluid intelligence, which was consistent with previous studies showing that the levels of fluid intelligence vary widely across populations (Finn et al., 2015; Kievit et al., 2016). Fluid intelligence reflects an individual’s innate ability to respond to complex situations, including the ability to process and learn new information and solve problems (Harada et al., 2013). The loss of fluid intelligence was associated with damage to restricted regions of the frontal and parietal cortices (Woolgar et al., 2010). The interactions between distributed brain regions during the movie are thought to reflect continuous processing aimed at extracting meaningful information from complex stimuli, which may support behavioral performance in day-to-day life (Hasson et al., 2010; Jaaskelainen et al., 2021). Combining the findings of the current study with the results of previous studies, these changes in ISFC between the CN and thalamic subdivisions might be associated with successful completion of cognitive processes.

We found decreased ISFC with age between the median and posterior thalamus and the DMN_C, which mainly contains the parahippocampal gyrus. Age-related decreases in ISFC were also found between the median thalamus and DMN_B, DMN_D, which mainly included the prefrontal cortex and the middle temporal lobe (MTL). Meanwhile, increased ISFC was found between the anterior thalamus and DMN_B and DMN_D, and between the ventral thalamus and DMN_A, which mainly consists of the PCC/precuneus. According to a review from Fama and Sullivan (2015), anterior thalamic nuclei and MD nuclei connect with the hippocampus and parahippocampal cortices to support information encoding and recollective processes (Fama and Sullivan, 2015). The ventromedial prefrontal cortex (vmPFC) and PCC, two core regions in the DMN, interact with specific brain systems to support different cognitive processes (Uddin et al., 2009). The vmPFC is implicated in decision-making and social cognition, and the PCC plays a critical role in self-relation information integration (Uddin et al., 2009; Hiser and Koenigs, 2018). Additionally, the DMN also contains the MTL subsystem (preferentially active when making a decision about the future) and dorsomedial prefrontal cortex (dmPFC) subsystem, which is active in the process of self-reference (Campbell et al., 2013). Using movie-watching state fMRI data, Geerligs et al. (2018) found decreased synchrony with age in the MTL, medial prefrontal cortex and frontoparietal network, which suggests age-related declines in memory and attentional control (Geerligs et al., 2018). Several structural and functional MRI studies have found that the DMN has extensive connections with thalamic subdivisions. Using diffusion tensor imaging, Behrens et al. (2003) found that the prefrontal cortex projects to the MD and VA nuclei, and several medial temporal regions project to parts of the anterior thalamus (Behrens et al., 2003). Cunningham et al. (2017) found structural connections between the precuneus/PCC and thalamic subdivisions that project to the parietal lobes, and the extent of connections decreases with age (Cunningham et al., 2017). Using resting-state fMRI, Yuan et al. (2016) found that the DMN is connected with the MD nucleus, pulvinar, AV nucleus and ventral thalamic nuclei (Yuan et al., 2016). The FC between the DMN and VP nucleus, pulvinar decreases with age, which is positively correlated with decreased cognitive flexibility (Steiner et al., 2020). Fair et al. (2010) found decreased FC between the temporal cortex and posterior and ventral subdivisions of the thalamus and increased FC between the frontal cortex and dorsal subdivisions of the thalamus during maturation (Fair et al., 2010). Moreover, several resting state and task-based fMRI studies have shown that the interactions between the prefrontal cortex and MD nucleus are critical for cognitive flexibility, as well as working memory maintenance (Halassa and Kastner, 2017; Rikhye et al., 2018). Using movie-watching state fMRI data, we found both increased and decreased ISFC with age between the DMN and thalamic subdivisions, and changed ISFC was positively correlated with decreased fluid intelligence. Using resting state fMRI, Wen et al. (2020) found that the local functional connectivity density of the left hippocampus showed a positive quadratic relationship with age, and Vij et al. (2018) found a positive quadratic relationship between the component volume of the DMN and age. In the present study, we found that the ISFC between the DMN_C and part of the lateral thalamus showed a positive quadratic relationship with age.

Meanwhile, we found age-related decreases in ISFC between the VP nucleus, pulvinar of the thalamus and the attention networks, VN, between medial and ventral portions of the thalamus and the SMN while watching movies. Decreased ISFC between the thalamus and these networks was positively correlated with decreased fluid intelligence with age. This is consistent with previous studies in which preferential integration of DAN and VN is observed in the pulvinar, suggesting that the pulvinar plays a critical role not only in attentional selection but also flexibly mediates the integration of visual information in the ever-changing context (Saalmann et al., 2012; Greene et al., 2020). Moreover, the interactions between the pulvinar and the visual and inferior temporal cortices are necessary for normal sensory and attentional processing (Zhou et al., 2016). Using resting-state fMRI, Steiner et al. (2020) found that the FC between the VL nucleus and the DAN is significantly correlated with attentional control performance (Steiner et al., 2020). From the perspective of functional connectivity, connectivity with the SMN is located in the ventral intermediate thalamus, corresponding to the VL nucleus and VP nucleus, and connectivity with the motor network is located in the VP nucleus and a small part of the MD nucleus (Yuan et al., 2016; Dong et al., 2021). In addition, several structural imaging studies have found that the VA and VL nuclei project to the premotor cortex and primary motor cortex, which supports control functions such as action (Behrens et al., 2003; de Bourbon-Teles et al., 2014). Using resting-state fMRI, Fair et al. (2010) found decreased FC between the medial thalamus and somatosensory cortex across development (Fair et al., 2010). Goldstone et al. (2018) revealed that higher FC between the thalamus and motor networks in older adults is associated with faster reaction times (Goldstone et al., 2018). Hence, decreased ISFC between the thalamus and attention networks, VN, SMN might suggest less attention to movie watching and worse abilities to process sensory information in older adults. Using resting state fMRI, Wen et al. (2020) found that the local functional connectivity density of the left precentral gyrus showed a positive quadratic relationship with age, while the local neural activity of the postcentral gyrus showed a negative quadratic relationship with age. Vij et al. (2018) also found a negative quadratic relationship between the component volume of the SMN and age. Using movie-watching state fMRI, we found that the ISFC between SMN_B and the medial thalamus showed a negative quadratic relationship with age, while the ISFC between SMN_B and the ventral thalamus showed a positive quadratic relationship with age.

Cognitive aging is a lifelong process, and cognitive decline in older adults may progress to mild cognitive impairment with high risk of developing dementia. Movie fMRI is emerging as a powerful tool to explore brain function in more realistic settings, which provides insights into our understanding of cognition (Eickhoff et al., 2020). Recently, accumulating studies have used movie fMRI to reveal cognitively related imaging biomarkers, thus contributing to the development of interventions to delay cognitive decline (Reiman et al., 2012; Campbell et al., 2015; Geerligs et al., 2018). Using an ISFC analysis on movie fMRI data, we found that functional interactions between distinct thalamic subregions and cortical networks were associated with age-related cognitive decline, providing further reference for delaying and intervening in cognitive aging.

Several limitations should be considered. First, because of the different sizes and locations of thalamic nuclei and the limitation of fMRI spatial resolution, it is difficult to accurately locate each thalamic nucleus with standardized templates. Hence, higher resolution fMRI data are needed. Second, behavioral indicators were relatively inadequate. Only fluid intelligence, which is related to executive control and reflects inner cognitive abilities, was assessed in our study. More behavioral data should be used to reveal the relationships between ISFCs and cognition. Furthermore, no direct measurement of behavioral outcomes was made during the movie watching. Third, the movie stimuli have less experimental control, so we cannot rule out the influence of personal interests on the thalamo-cortical network ISFCs across the lifespan, such as the gaze position of participants in a particular scene. Fourth, the number of clinicians involved in the process of assessing the behavioral outcome was unclear. These variabilities, such as the experience and knowledge of the interviewers might influence the results of the behavioral test associated with the brain functions of subjects. Finally, functional communications between brain regions are considered to change over time, while dynamic connectivity may be a complementary approach to characterize the functions of brain regions (Di and Biswal, 2020). Hence, longitudinal studies are required to further understand the temporal evolution of thalamo-cortical network development and its relationship with cognition.

## Conclusion

Using an ISFC analysis, we found age-related changes in functional correlations between the thalamus and cortical networks. Meanwhile, the alterations in ISFC were associated with declines in cognitive function across the adult lifespan. Collectively, our findings demonstrate that the interactions between different thalamic nuclei and cortical networks play a critical role in cognitive processes, providing a further reference for delaying and intervening in cognitive aging. Meanwhile, the ISFC analysis is an effective way to identify the stimulus-induced brain dynamics under natural stimulation.

## Data availability statement

The original contributions presented in this study are included in the article/Supplementary material, further inquiries can be directed to the corresponding author.

## Ethics statement

The studies involving human participants were reviewed and approved by the Cambridgeshire 2 Research Ethics Committee. The patients/participants provided their written informed consent to participate in this study.

## Author contributions

WC and QJ designed the study. WC and JN wrote the first draft of the manuscript. JN, ZZ, ZW, LX, and QM performed the data analysis. XZ, LK, and SW advised on statistical analysis. LD, JQ, QJ, and WC drafted the manuscript and revised it critically for important intellectual content. All authors read and approved the final manuscript.

## Footnotes

1
http://www.cam-can.org/

2
https://www.fil.ion.ucl.ac.uk/spm/software/spm12

3
http://www.neuro.uestc.edu.cn/NIT.html
