# Supplementary material for: Thalamo-cortical inter-subject functional correlation during movie watching across the adult lifespan
Source: Front Neurosci. 2022 Sep 21;16:984571. doi: 10.3389/fnins.2022.984571 (PMC9534554; doi:10.3389/fnins.2022.984571)
Supplement: Supplementary file 1 [file Table_1.DOCX]

***Supplementary Material***

**Supplementary Table 1.** Demographic data and cognitive measures of all participants.

| Variables |  |
| --- | --- |
| Age range (years) | 18-87 (51.75 ± 17.26) |
| Number | 500 |
| Gender (male/female) | 248/252 |
| Education |  |
| University | 486 |
| A’ levels | 6 |
| GCSE grade | 3 |
| None > 16 | 3 |
| MMSE | 29.07 ± 1.17 |
| Fluid intelligence | 32.79 ± 6.39 |

The education is defined as the age of completing full-time education. MMSE and fluid intelligence are expressed as Mean ± Standard deviation.

Abbreviation: MMSE, Mini-Mental State Exam.

**Supplementary Table 2.** The brain regions contained in 17 cortical networks.

| Networks | Regions | MNI coordinates | | |
| --- | --- | --- | --- | --- |
|  |  | X | Y | Z |
| VPN | FuG_L_3_2 | -31 | -64 | -14 |
|  | FuG_R_3_2 | 31 | -62 | -14 |
|  | MVOcC_L_5_1 | -11 | -82 | -11 |
|  | MVOcC_R_5_1 | 10 | -85 | -9 |
|  | MVOcC_L_5_3 | -6 | -94 | 1 |
|  | MVOcC_R_5_3 | 8 | -90 | 12 |
|  | MVOcC_R_5_4 | 18 | -60 | -7 |
|  | LOcC_L_4_1 | -31 | -89 | 11 |
|  | LOcC_R_4_1 | 34 | -86 | 11 |
|  | LOcC_L_4_3 | -18 | -99 | 2 |
|  | LOcC_R_4_3 | 22 | -97 | 4 |
|  | LOcC_L_4_4 | -30 | -88 | -12 |
|  | LOcC_R_4_4 | 32 | -85 | -12 |
|  | LOcC_L_2_2 | -22 | -77 | 36 |
| VCN | PhG_L_6_6 | -17 | -39 | -10 |
|  | PhG_R_6_6 | 19 | -36 | -11 |
|  | PCun_L_4_3 | -12 | -67 | 25 |
|  | PCun_R_4_3 | 16 | -64 | 25 |
|  | MVOcC_L_5_2 | -5 | -81 | 10 |
|  | MVOcC_R_5_2 | 7 | -76 | 11 |
|  | MVOcC_L_5_4 | -17 | -60 | -6 |
|  | MVOcC_L_5_5 | -13 | -68 | 12 |
|  | MVOcC_R_5_5 | 15 | -63 | 12 |
|  | LOcC_L_2_1 | -11 | -88 | 31 |
|  | LOcC_R_2_1 | 16 | -85 | 34 |
| SMN_A | SFG_L_7_5 | -6 | -5 | 58 |
|  | SFG_R_7_5 | 7 | -4 | 60 |
|  | PrG_L_6_3 | -26 | -25 | 63 |
|  | PrG_R_6_3 | 34 | -19 | 59 |
|  | PrG_L_6_4 | -13 | -20 | 73 |
|  | PrG_R_6_4 | 15 | -22 | 71 |
|  | PCL_R_2_1 | 10 | -34 | 54 |
|  | PCL_L_2_2 | -4 | -23 | 61 |
|  | PCL_R_2_2 | 5 | -21 | 61 |
|  | SPL_L_5_4 | -22 | -47 | 65 |
|  | SPL_R_5_4 | 23 | -43 | 67 |
|  | PCun_L_4_2 | -8 | -47 | 57 |
|  | PoG_R_4_3 | 48 | -24 | 48 |
|  | PoG_L_4_4 | -21 | -35 | 68 |
|  | PoG_R_4_4 | 20 | -33 | 69 |
| SMN_B | PrG_L_6_1 | -49 | -8 | 39 |
|  | PrG_R_6_1 | 55 | -2 | 33 |
|  | STG_L_6_2 | -54 | -32 | 12 |
|  | STG_R_6_2 | 54 | -24 | 11 |
|  | STG_L_6_3 | -50 | -11 | 1 |
|  | STG_R_6_3 | 51 | -4 | -1 |
|  | STG_R_6_4 | 66 | -20 | 6 |
|  | IPL_L_6_6 | -53 | -31 | 23 |
|  | IPL_R_6_6 | 55 | -26 | 26 |
|  | PoG_L_4_1 | -50 | -16 | 43 |
|  | PoG_R_4_1 | 50 | -14 | 44 |
|  | PoG_L_4_2 | -56 | -14 | 16 |
|  | PoG_R_4_2 | 56 | -10 | 15 |
|  | INS_L_6_1 | -36 | -20 | 10 |
|  | INS_R_6_1 | 37 | -18 | 8 |
|  | INS_L_6_5 | -38 | -8 | 8 |
|  | INS_R_6_5 | 39 | -7 | 8 |
| DAN_A | ITG_L_7_2 | -51 | -57 | -15 |
|  | ITG_R_7_2 | 53 | -52 | -18 |
|  | ITG_R_7_5 | 54 | -57 | -8 |
|  | FuG_L_3_3 | -42 | -51 | -17 |
|  | FuG_R_3_3 | 43 | -49 | -19 |
|  | SPL_L_5_2 | -15 | -71 | 52 |
|  | SPL_R_5_2 | 19 | -69 | 54 |
|  | SPL_L_5_5 | -27 | -59 | 54 |
|  | SPL_R_5_5 | 31 | -54 | 53 |
|  | IPL_L_6_1 | -34 | -80 | 29 |
|  | IPL_R_6_1 | 45 | -71 | 20 |
|  | LOcC_L_4_2 | -46 | -74 | 3 |
|  | LOcC_R_4_2 | 48 | -70 | -1 |
|  | LOcC_R_2_2 | 29 | -75 | 36 |
| DAN_B | SFG_L_7_4 | -18 | -1 | 65 |
|  | SFG_R_7_4 | 20 | 4 | 64 |
|  | MFG_L_7_6 | -32 | 4 | 55 |
|  | PrG_L_6_2 | -32 | -9 | 58 |
|  | PrG_R_6_2 | 33 | -7 | 57 |
|  | MTG_L_4_3 | -59 | -58 | 4 |
|  | MTG_R_4_3 | 60 | -53 | 3 |
|  | SPL_L_5_1 | -16 | -60 | 63 |
|  | SPL_R_5_1 | 19 | -57 | 65 |
|  | SPL_L_5_3 | -33 | -47 | 50 |
|  | SPL_R_5_3 | 35 | -42 | 54 |
|  | IPL_L_6_3 | -51 | -33 | 42 |
|  | IPL_R_6_3 | 47 | -35 | 45 |
|  | PCun_R_4_2 | 7 | -47 | 58 |
|  | PoG_L_4_3 | -46 | -30 | 50 |
| VAN | IFG_L_6_5 | -39 | 23 | 4 |
|  | PrG_L_6_5 | -52 | 0 | 8 |
|  | PrG_R_6_5 | 54 | 4 | 9 |
|  | PCL_L_2_1 | -8 | -38 | 58 |
|  | INS_L_6_4 | -38 | -4 | -9 |
|  | INS_R_6_4 | 39 | -2 | -9 |
|  | INS_L_6_6 | -38 | 5 | 5 |
|  | INS_R_6_6 | 38 | 5 | 5 |
|  | CG_L_7_5 | -5 | 7 | 37 |
|  | CG_R_7_5 | 4 | 6 | 38 |
|  | CG_R_7_6 | 6 | -20 | 40 |
| SN | SFG_R_7_1 | 7 | 16 | 54 |
|  | MFG_L_7_1 | -27 | 43 | 31 |
|  | MFG_R_7_4 | 42 | 44 | 14 |
|  | IFG_R_6_4 | 51 | 36 | -1 |
|  | IFG_R_6_5 | 42 | 22 | 3 |
|  | IFG_L_6_6 | -52 | 13 | 6 |
|  | IFG_R_6_6 | 54 | 14 | 11 |
|  | INS_R_6_2 | 33 | 14 | -13 |
|  | INS_L_6_3 | -34 | 18 | 1 |
|  | INS_R_6_3 | 36 | 18 | 1 |
|  | CG_R_7_3 | 5 | 28 | 27 |
|  | CG_L_7_6 | -7 | -23 | 41 |
| Limbic_A | STG_L_6_1 | -32 | 14 | -34 |
|  | STG_R_6_1 | 31 | 15 | -34 |
|  | STG_L_6_5 | -45 | 11 | -20 |
|  | STG_R_6_5 | 47 | 12 | -20 |
|  | ITG_L_7_1 | -45 | -26 | -27 |
|  | ITG_R_7_1 | 46 | -14 | -33 |
|  | ITG_L_7_3 | -43 | -2 | -41 |
|  | ITG_R_7_3 | 40 | 0 | -43 |
|  | ITG_L_7_7 | -55 | -31 | -27 |
|  | ITG_R_7_7 | 54 | -31 | -26 |
|  | FuG_L_3_1 | -33 | -16 | -32 |
|  | FuG_R_3_1 | 33 | -15 | -34 |
|  | PhG_L_6_1 | -27 | -7 | -34 |
|  | PhG_R_6_1 | 28 | -8 | -33 |
|  | PhG_L_6_4 | -19 | -12 | -30 |
|  | PhG_R_6_4 | 19 | -10 | -30 |
|  | PhG_L_6_5 | -23 | 2 | -32 |
|  | PhG_R_6_5 | 22 | 1 | -36 |
| Limbic_B | MFG_L_7_7 | -26 | 60 | -6 |
|  | OrG_L_6_3 | -23 | 38 | -18 |
|  | OrG_L_6_4 | -6 | 52 | -19 |
|  | OrG_R_6_4 | 6 | 57 | -16 |
|  | OrG_L_6_5 | -10 | 18 | -19 |
|  | OrG_R_6_5 | 9 | 20 | -19 |
| CN_A | MFG_L_7_2 | -42 | 13 | 36 |
|  | MFG_R_7_2 | 42 | 11 | 39 |
|  | MFG_L_7_4 | -41 | 41 | 16 |
|  | MFG_R_7_6 | 34 | 8 | 54 |
|  | IFG_L_6_1 | -46 | 13 | 24 |
|  | IFG_R_6_1 | 45 | 16 | 25 |
|  | IFG_L_6_2 | -47 | 32 | 14 |
|  | IFG_R_6_2 | 48 | 35 | 13 |
|  | OrG_R_6_3 | 23 | 36 | -18 |
|  | PrG_L_6_6 | -49 | 5 | 30 |
|  | PrG_R_6_6 | 51 | 7 | 30 |
|  | ITG_L_7_5 | -55 | -60 | -6 |
|  | IPL_L_6_2 | -38 | -61 | 46 |
|  | IPL_R_6_2 | 39 | -65 | 44 |
| CN_B | SFG_R_7_2 | 22 | 26 | 51 |
|  | SFG_L_7_6 | -5 | 36 | 38 |
|  | SFG_R_7_6 | 6 | 38 | 35 |
|  | MFG_L_7_3 | -28 | 56 | 12 |
|  | MFG_R_7_3 | 28 | 55 | 17 |
|  | MFG_R_7_5 | 42 | 27 | 39 |
|  | MFG_R_7_7 | 25 | 61 | -4 |
|  | MTG_R_4_1 | 65 | -29 | -13 |
|  | ITG_L_7_6 | -59 | -42 | -16 |
|  | ITG_R_7_6 | 61 | -40 | -17 |
|  | IPL_R_6_4 | 57 | -44 | 38 |
| CN_C | MFG_R_7_1 | 30 | 37 | 36 |
|  | PCun_L_4_1 | -5 | -63 | 51 |
|  | PCun_R_4_1 | 6 | -65 | 51 |
| DMN_A | SFG_L_7_2 | -18 | 24 | 53 |
|  | SFG_L_7_7 | -8 | 56 | 15 |
|  | SFG_R_7_7 | 8 | 58 | 13 |
|  | MFG_L_7_5 | -33 | 23 | 45 |
|  | OrG_L_6_1 | -7 | 54 | -7 |
|  | OrG_R_6_1 | 6 | 47 | -7 |
|  | IPL_R_6_5 | 53 | -54 | 25 |
|  | PCun_L_4_4 | -6 | -55 | 34 |
|  | PCun_R_4_4 | 6 | -54 | 35 |
|  | CG_L_7_1 | -4 | -39 | 31 |
|  | CG_R_7_1 | 4 | -37 | 32 |
|  | CG_L_7_3 | -6 | 34 | 21 |
|  | CG_L_7_7 | -4 | 39 | -2 |
|  | CG_R_7_7 | 5 | 41 | 6 |
| DMN_B | SFG_L_7_1 | -5 | 15 | 54 |
|  | SFG_L_7_3 | -11 | 49 | 40 |
|  | SFG_R_7_3 | 13 | 48 | 40 |
|  | IFG_L_6_3 | -53 | 23 | 11 |
|  | IFG_R_6_3 | 54 | 24 | 12 |
|  | IFG_L_6_4 | -49 | 36 | -3 |
|  | OrG_L_6_2 | -36 | 33 | -16 |
|  | OrG_R_6_2 | 40 | 39 | -14 |
|  | OrG_L_6_6 | -41 | 32 | -9 |
|  | OrG_R_6_6 | 42 | 31 | -9 |
|  | STG_R_6_6 | 56 | -12 | -5 |
|  | MTG_L_4_1 | -65 | -30 | -12 |
|  | MTG_L_4_2 | -53 | 2 | -30 |
|  | MTG_R_4_2 | 51 | 6 | -32 |
|  | MTG_L_4_4 | -58 | -20 | -9 |
|  | ITG_L_7_4 | -56 | -16 | -28 |
|  | ITG_R_7_4 | 55 | -11 | -32 |
|  | IPL_L_6_4 | -56 | -49 | 38 |
| DMN_C | PhG_L_6_2 | -25 | -25 | -26 |
|  | PhG_R_6_2 | 26 | -23 | -27 |
|  | PhG_L_6_3 | -28 | -32 | -18 |
|  | PhG_R_6_3 | 30 | -30 | -18 |
|  | CG_L_7_4 | -8 | -47 | 10 |
|  | CG_R_7_4 | 9 | -44 | 11 |
| DMN_D | STG_L_6_4 | -62 | -33 | 7 |
|  | STG_L_6_6 | -55 | -3 | -10 |
|  | MTG_R_4_4 | 58 | -16 | -10 |
|  | pSTS_L_2_1 | -54 | -40 | 4 |
|  | pSTS_R_2_1 | 53 | -37 | 3 |
|  | pSTS_L_2_2 | -52 | -50 | 11 |
|  | pSTS_R_2_2 | 57 | -40 | 12 |
|  | IPL_L_6_5 | -47 | -65 | 26 |

Abbreviation: VPN, visual peripheral network; VCN, visual central network; SMN_A, sensorimotor network A; SMN_B, sensorimotor network B; DAN_A, dorsal attention network A; DAN_B, dorsal attention network B; VAN, ventral attention network; SN, salience network; Limbic_A, limbic network A; Limbic_B, limbic network B; CN_A, control network A; CN_B, control network B; CN_C, control network C; DMN_A, default mode network A; DMN_B, default mode network B; DMN_C, default mode network C; DMN_D, default mode network D; SFG, superior frontal gyrus; MFG, middle frontal gyrus; IFG, inferior frontal gyrus; OrG, orbital gyrus; PrG, precentral gyrus; PCL, paracentral lobule; STG, superior temporal gyrus; MTG, middle temporal gyrus; ITG, inferior temporal gyrus; FuG, fusiform gyrus; PhG, parahippocampal gyrus; pSTS, posterior superior temporal sulcus; SPL, superior parietal lobule; IPL, inferior parietal lobule; PCun, precuneus; PoG, postcentral gyrus; INS, insular gyrus; CG, cingulate gyrus; MVOcC, medio ventral occipital cortex; LOcC, lateral occipital cortex; L, left; R, right.

**Supplementary Table 3.** Demographic data and cognitive measures of participants in split-half validation analysis.

| Group | 1 | 2 | 3 | 4 | 5 | 6 | 7 |
| --- | --- | --- | --- | --- | --- | --- | --- |
| Age range(years) | 18-27 | 28-37 | 38-47 | 48-57 | 58-67 | 68-77 | 78-87 |
| Number | 17 | 48 | 51 | 44 | 28 | 36 | 26 |
| Male/female | 7/10 | 23/25 | 30/21 | 25/19 | 15/13 | 18/18 | 14/12 |
| Education(years) | 21.35±2.52 | 21.90±2.64 | 22.02±3.88 | 20.30±3.27 | 20.50±4.18 | 19.26±4.62 | 19.27±3.63 |
| MMSE | 29.47±1.01 | 29.52±0.85 | 29.12±1.11 | 29.23±0.94 | 28.93±1.25 | 28.66±1.30 | 28.31±1.72 |
| Fluid intelligence | 38.06±3.96 | 36.69±4.26 | 35.57±4.83 | 32.84±4.01 | 30.36±5.85 | 27.19±6.12 | 23.35±5.74 |

The education is defined as the age of completing full-time education. Education, MMSE and fluid intelligence are expressed as Mean ± Standard deviation.

Abbreviation: MMSE, Mini-Mental State Exam.


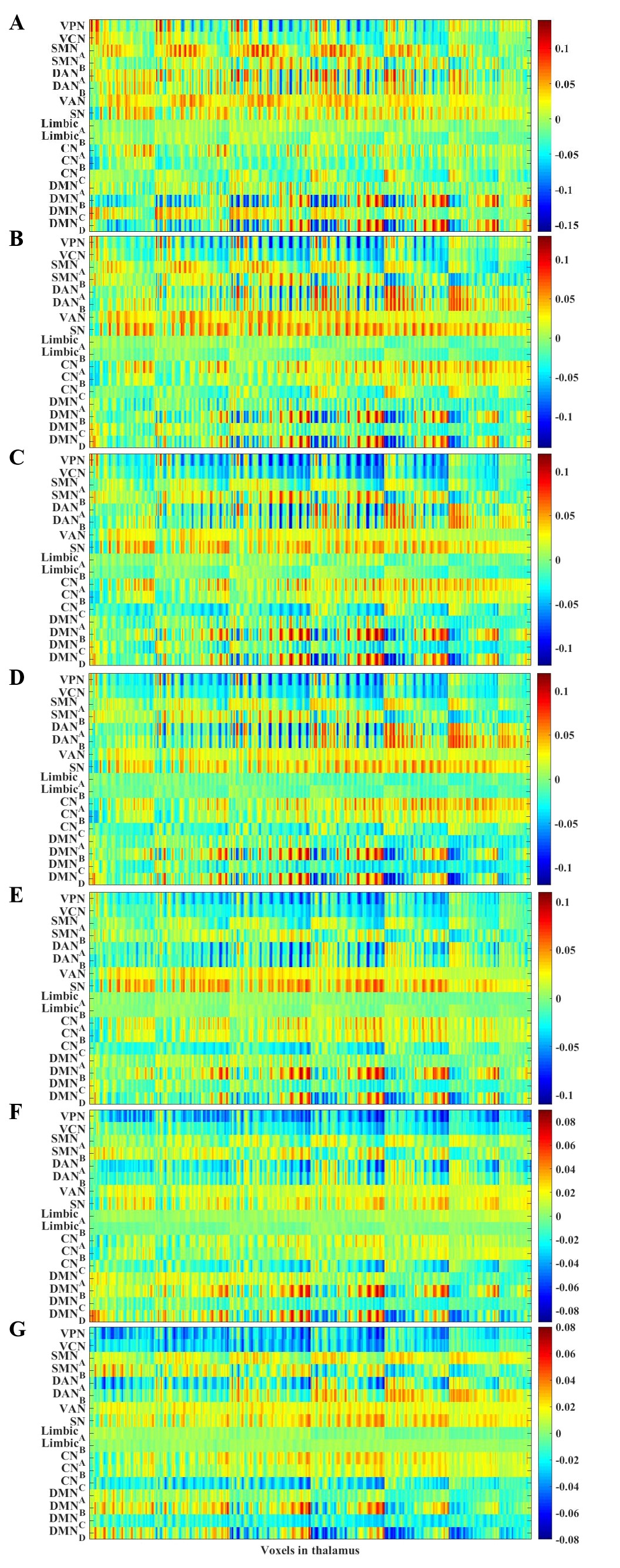


**Supplementary Figure 1.** Mean ISFC matrices of each subgroup. A. 18-27 years. B. 28-37 years. C. 38-47 years. D. 48-57 years. E. 58-67 years. F. 68-77 years. G. 78-87 years. The colorbar represents the mean correlation coefficient. Abbreviation: VPN, visual peripheral network; VCN, visual central network; SMN_A, sensorimotor network A; SMN_B, sensorimotor network B; DAN_A, dorsal attention network A; DAN_B, dorsal attention network B; VAN, ventral attention network; SN, salience network; Limbic_A, limbic network A; CN_A, control network A; CN_B, control network B; CN_C, control network C; DMN_A, default mode network A; DMN_B, default mode network B; DMN_C, default mode network C; DMN_D, default mode network D.


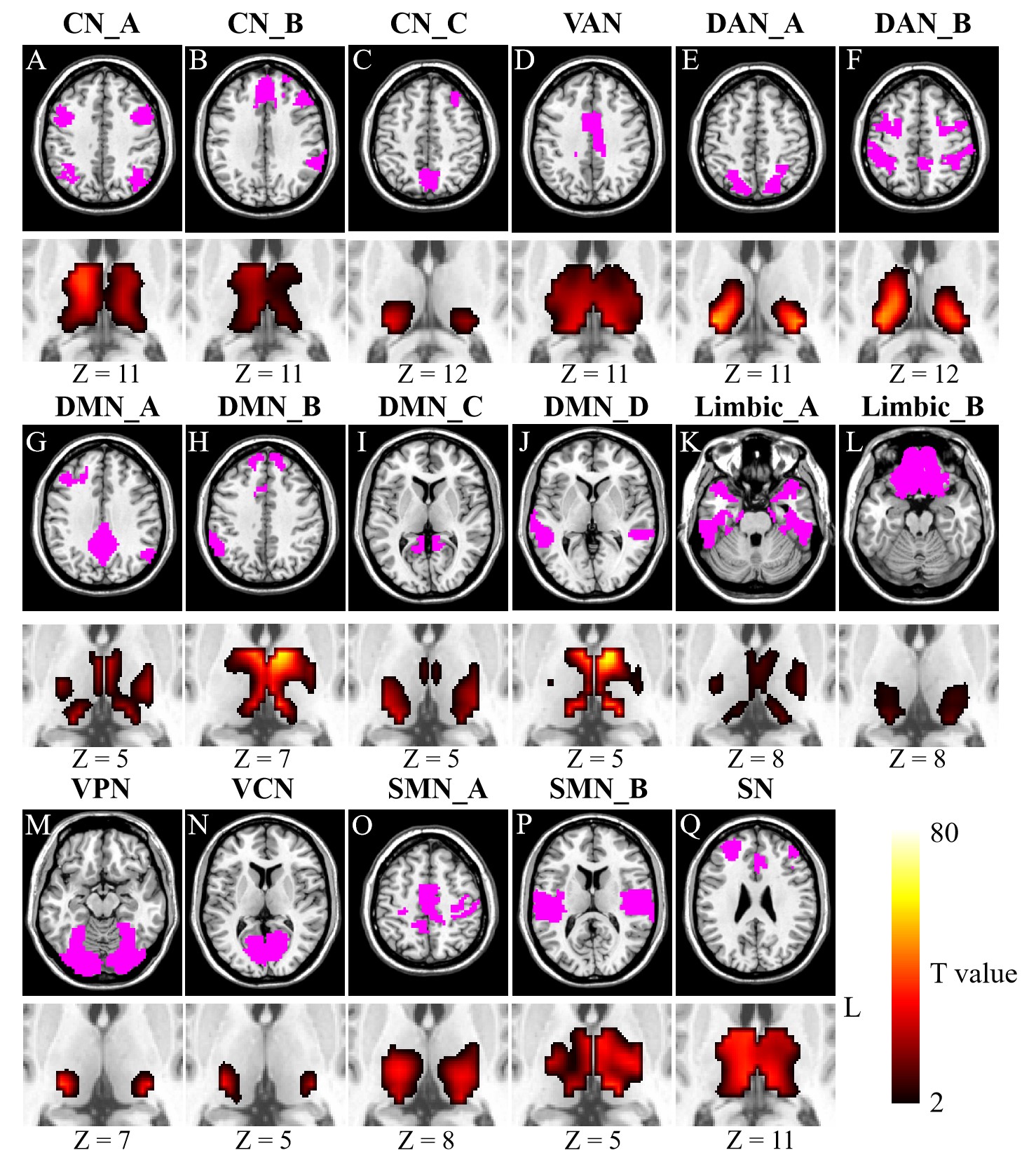


**Supplementary Figure 2.** The significant ISFCs between the thalamus and each cortical network across all participants (one sample t-test with FDR correction *p* < 0.05). The colorbar represents the T values. L represents the left hemisphere. Abbreviation: CN_A, control network A; CN_B, control network B; CN_C, control network C; VAN, ventral attention network; DAN_A, dorsal attention network A; DAN_B, dorsal attention network B; DMN_A, default mode network A; DMN_B, default mode network B; DMN_C, default mode network C; DMN_D, default mode network D; Limbic_A, limbic network A; Limbic_B, limbic network B; VPN, visual peripheral network; VCN, visual central network; SMN_A, sensorimotor network A; SMN_B, sensorimotor network B; SN, salience network.

1. **Effect of grouping on the relationship between ISFC and age, fluid intelligence.**

To investigate the influence of grouping on ISFC analysis, we also grouped all participants by different age range and performed ISFC analysis and GLM analysis respectively, including 5 years, 15 years, 20 years, 25 years and 30 years. To be specific, the mean time courses of each brain network (17 networks in total) and time courses of thalamus (620 voxels in total) were extracted for each participant. All participants were divided into 14 groups with an age range of 5 years, 7 groups with an age range of 10 years, 5 groups with an age range of 15 years, 4 groups with an age range of 20 years, 3 groups with an age range of 25 years, 3 groups with an age range of 30 years. If there are n subjects in each group, Pearson correlation was computed between the mean time courses of each brain network of one subject and time courses of the thalamus of all other subjects, resulting in n-1 ISFC matrix (17 × 620) for each subject. These matrices were averaged and performed Fisher z transformation to obtain an ISFC matrix for each subject. Then, GLM analysis was performed respectively under different grouping conditions to investigate the relation between ISFC and age, fluid intelligence.


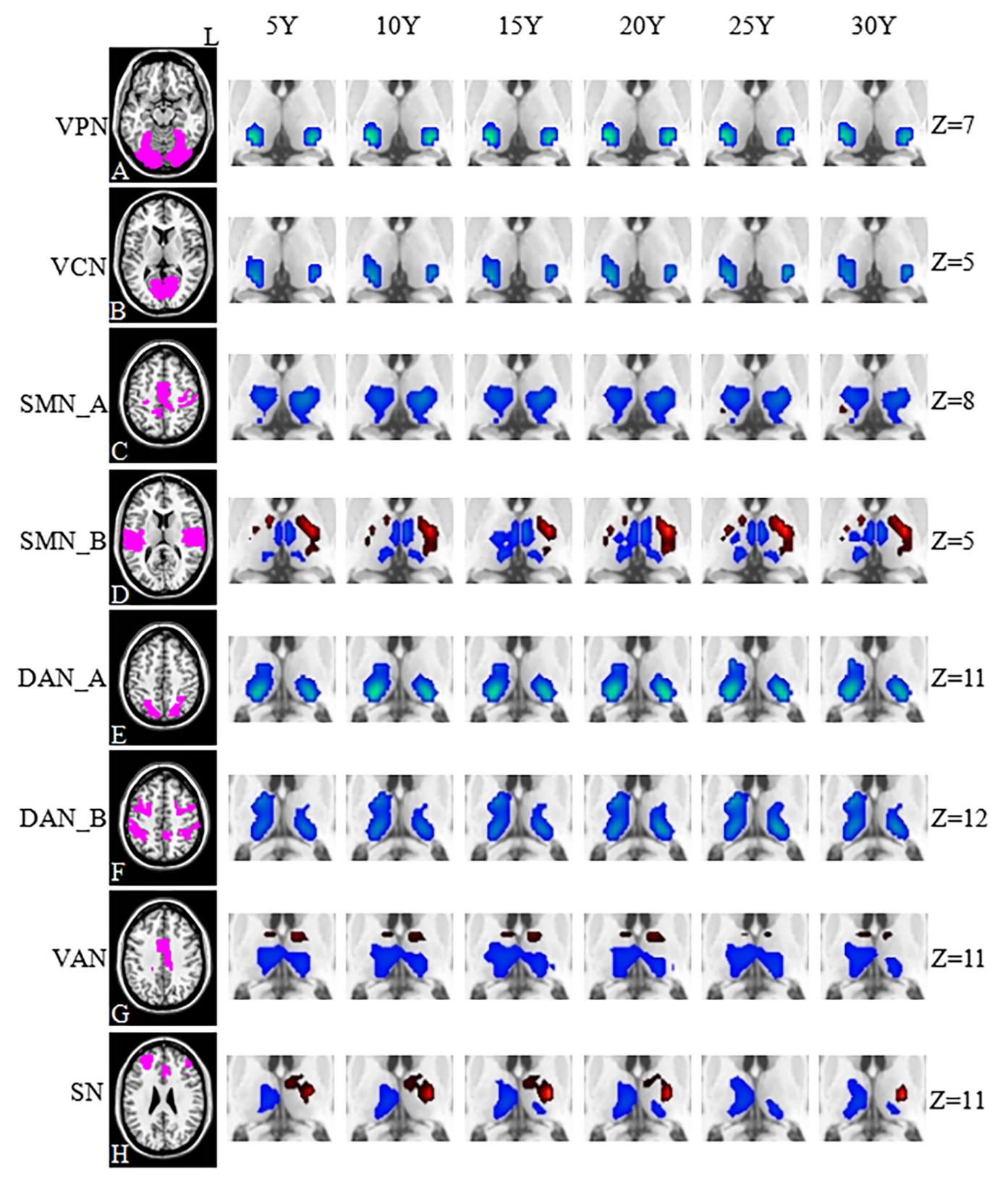


**
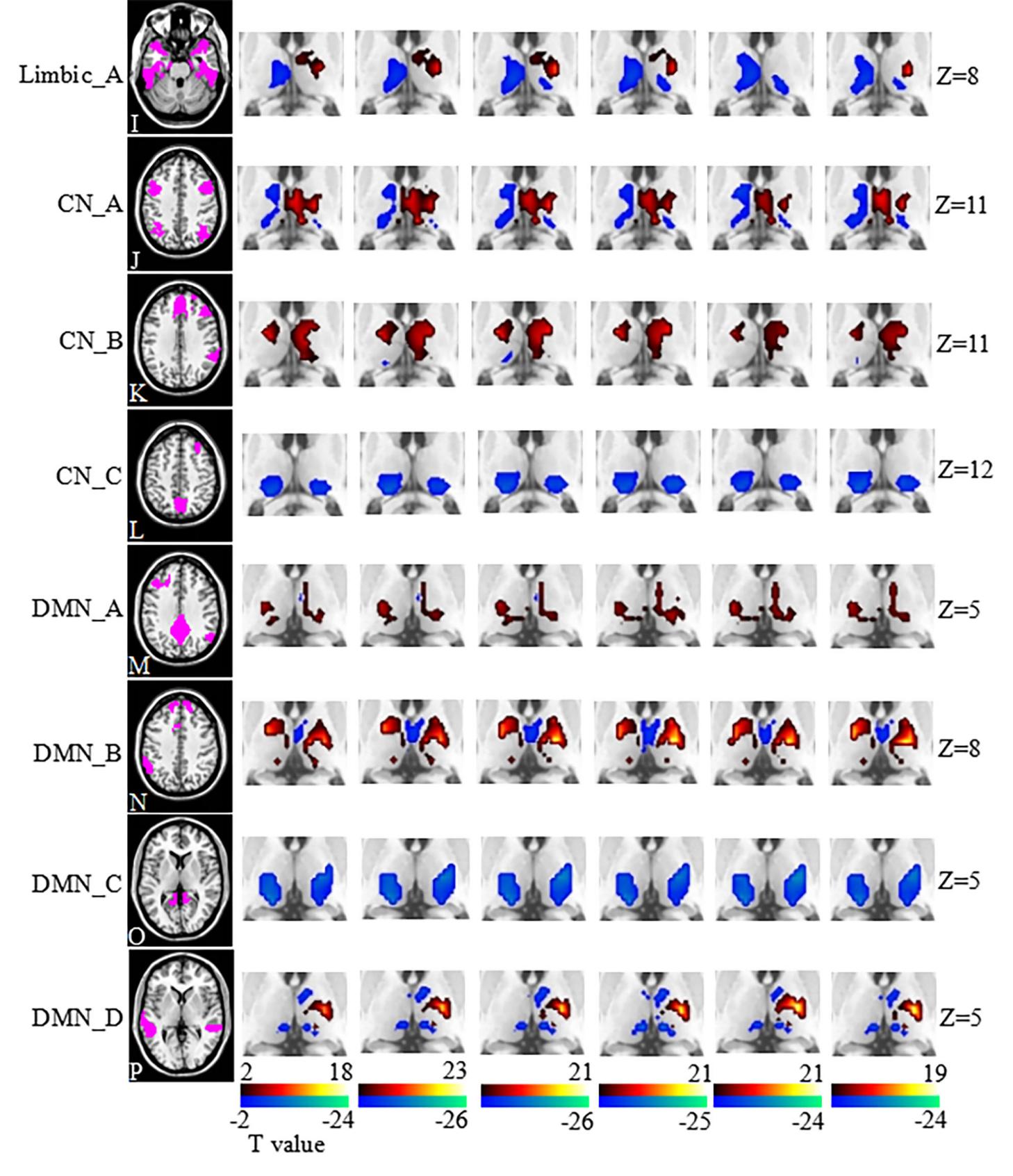
**

**Supplementary Figure 3.** Age-related changes of ISFCs that were calculated with different ranges of age (columns) between thalamus and cortical networks (rows A through P). Color bar signifies the T statistics (warm color, ISFCs increased significantly as age increased; cool color, ISFCs decreased significantly as age increased; FDR-corrected). L represents the left hemisphere. A minimum cluster size was 23 adjacent voxels. Abbreviation: VPN, visual peripheral network; VCN, visual central network; SMN_A, sensorimotor network A; SMN_B, sensorimotor network B; DAN_A, dorsal attention network A; DAN_B, dorsal attention network B; VAN, ventral attention network; SN, salience network; Limbic_A, limbic network A; CN_A, control network A; CN_B, control network B; CN_C, control network C; DMN_A, default mode network A; DMN_B, default mode network B; DMN_C, default mode network C; DMN_D, default mode network D.


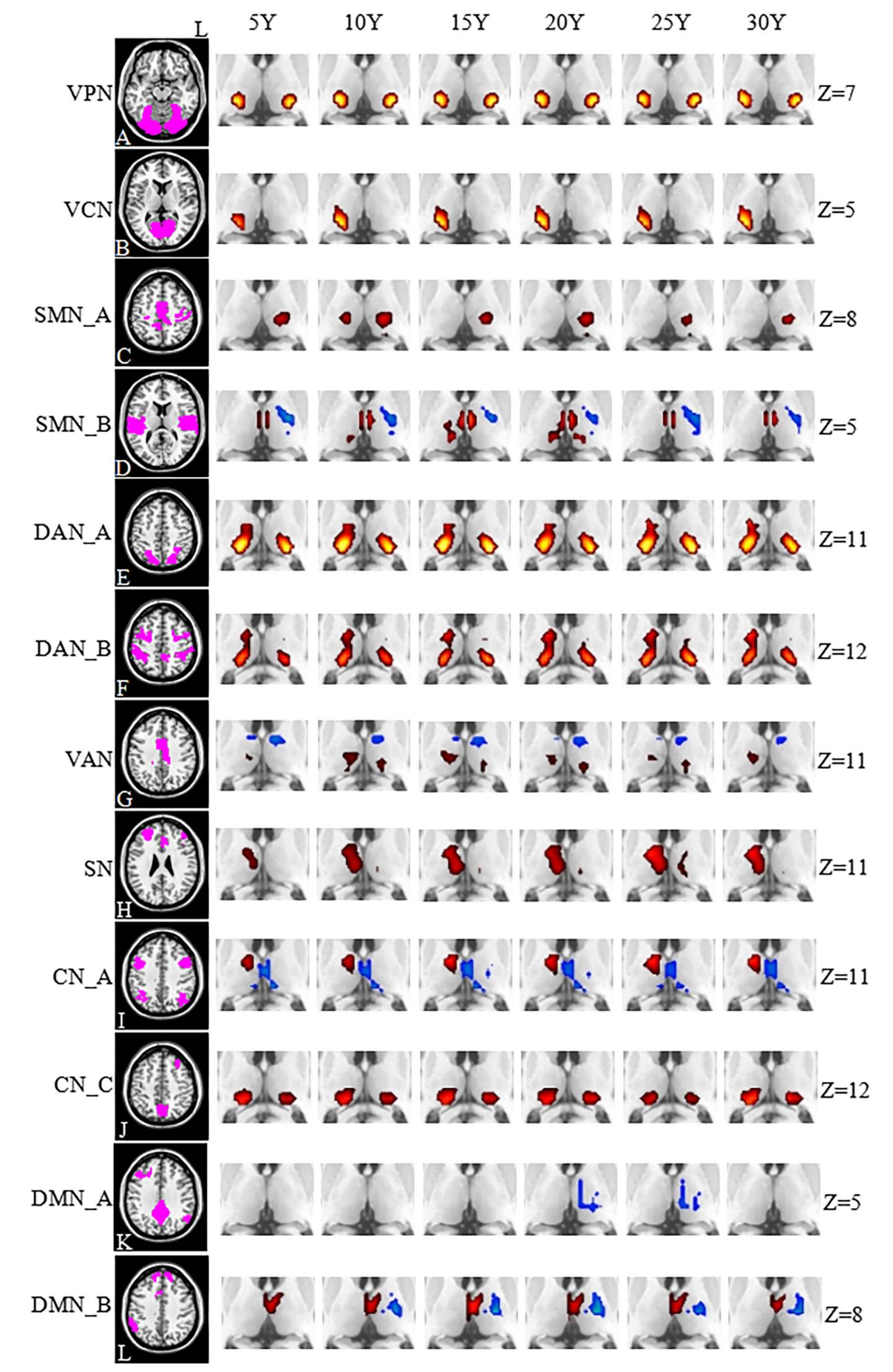


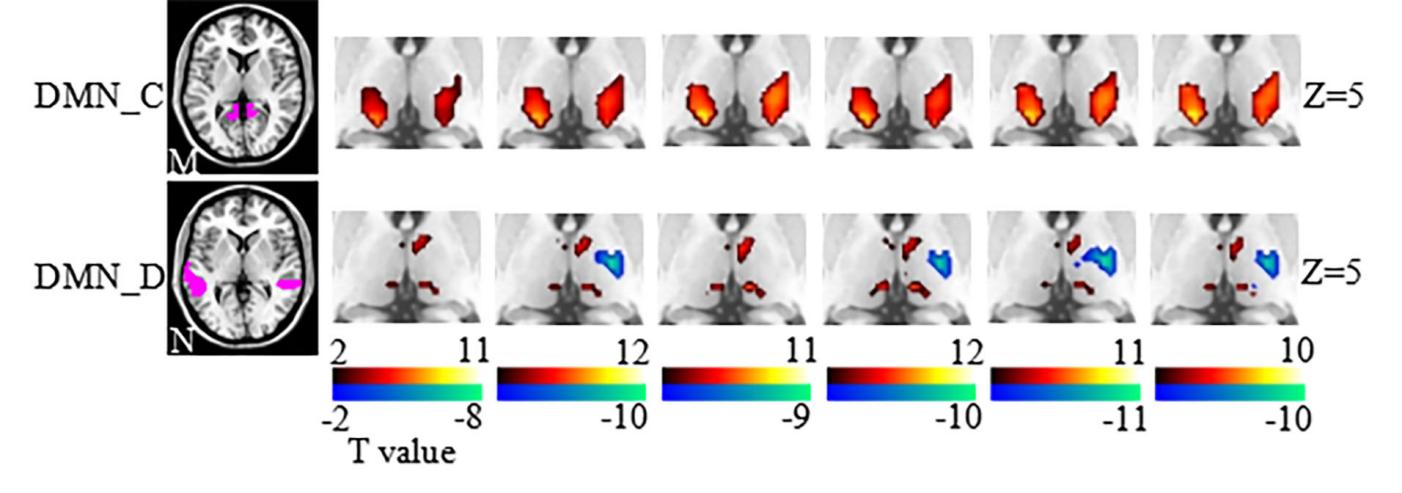


**Supplementary Figure 4.** Relationships between fluid intelligence and ISFCs that were calculated with different ranges of age (columns) between thalamus and cortical networks (rows A through N). Color bar signifies the T statistics (warm color, ISFCs increased significantly as fluid intelligence scores increased; cool color, ISFCs decreased significantly as fluid intelligence scores increased; FDR-corrected). L represents the left hemisphere. A minimum cluster size was 23 adjacent voxels. Abbreviation: VPN, visual peripheral network; VCN, visual central network; SMN_A, sensorimotor network A; SMN_B, sensorimotor network B; DAN_A, dorsal attention network A; DAN_B, dorsal attention network B; VAN, ventral attention network; SN, salience network; CN_A, control network A; CN_C, control network C; DMN_A, default mode network A; DMN_B, default mode network B; DMN_C, default mode network C; DMN_D, default mode network D.

1. **Quadratic relationship between the ISFC of thalamo-cortical networks and age.**

To investigate the quadratic relationship between age and the ISFC of thalamo-cortical networks. GLM was performed while adding the gender, education, head motion (mFD) and brain volume as covariates (FDR correction with *p* < 0.05).

$$ISFC = \beta_{0}+ \beta_{1} \cdot age + \beta_{2} \cdot{age}^{2}+ \beta_{3} \cdot sex + \beta_{4} \cdot education + \beta_{5} \cdot mFD+ \beta_{6} \cdot volume$$

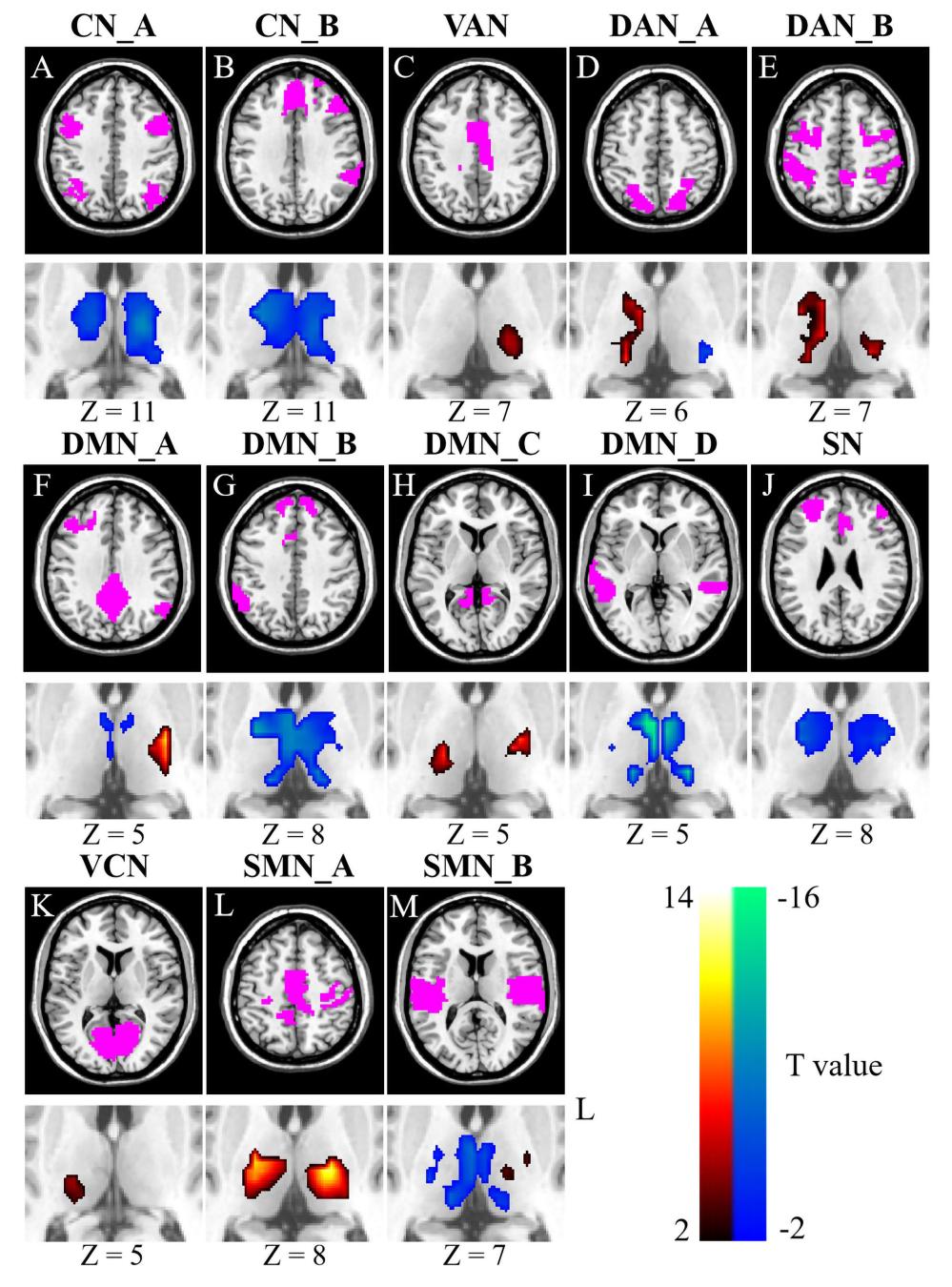


**Supplementary Figure 5.** Relationships between age^2^ and ISFCs between the thalamus and cortical networks (A through M). Color bar signifies the T values (warm color, ISFCs increased significantly as age increased; cool color, ISFCs decreased significantly as age increased; FDR-corrected). L represents the left hemisphere. A minimum cluster size was 23 adjacent voxels. Abbreviation: CN_A, control network A; CN_B, control network B; VAN, ventral attention network; DAN_A, dorsal attention network A; DAN_B, dorsal attention network B; DMN_A, default mode network A; DMN_B, default mode network B; DMN_C, default mode network C; DMN_D, default mode network D; SN, salience network; VCN, visual central network; SMN_A, sensorimotor network A; SMN_B, sensorimotor network B.

1. **Relationships of ISFC between the thalamus and 7 cortical networks with age, fluid intelligence.**

We also investigated age-related changes in ISFC of thalamo-corical networks and its relationship with fluid intelligence with 7 cortical brain networks that from Yeo et al. The mean time courses of each brain network and time courses of thalamus (620 voxels in total) were extracted for each participant. The ISFC matrix (7× 620) was then calculated for each participant within each group. GLM analyses were performed to investigate the relationship between the ISFC and age, fluid intelligence.


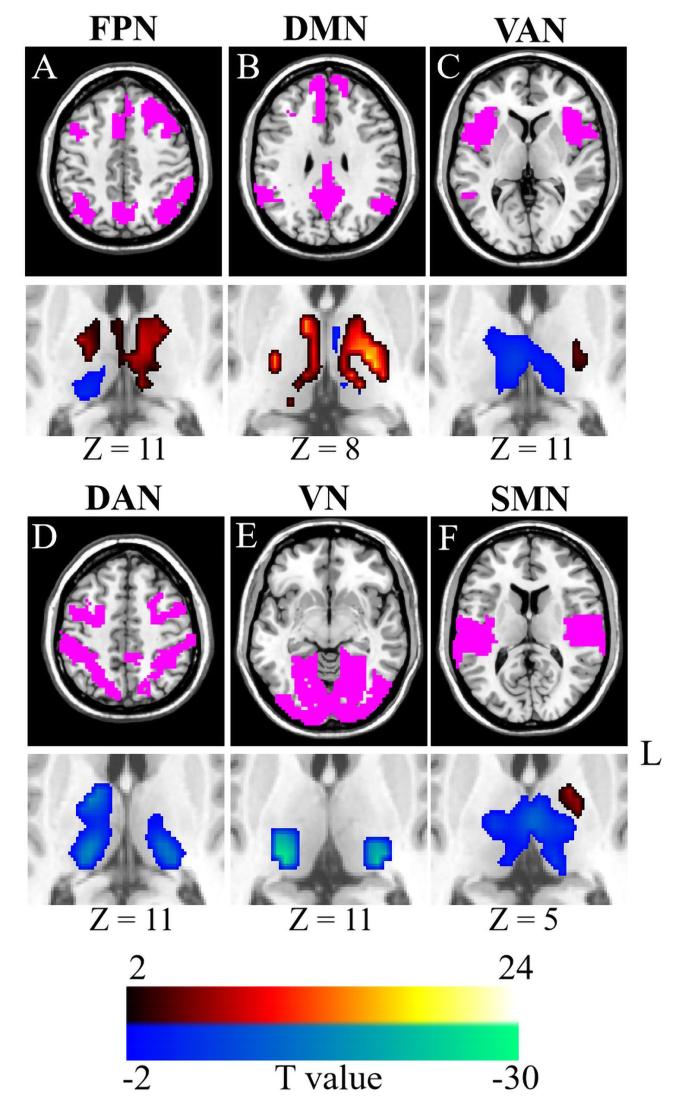


**Supplementary Figure 6.** Age-related changes of ISFCs between the thalamus and 7 cortical networks (A through F, 7 cortical networks that were defined by Yeo et al.). Color bar signifies the T values (warm color, ISFCs increased significantly as age increased; cool color, ISFCs decreased significantly as age increased; FDR-corrected). L represents the left hemisphere. A minimum cluster size was 23 adjacent voxels. Abbreviation: FPN, frontoparietal network; DMN, default mode network; VAN, ventral attention network; DAN, dorsal attention network; VN, visual network; SMN, sensorimotor network.


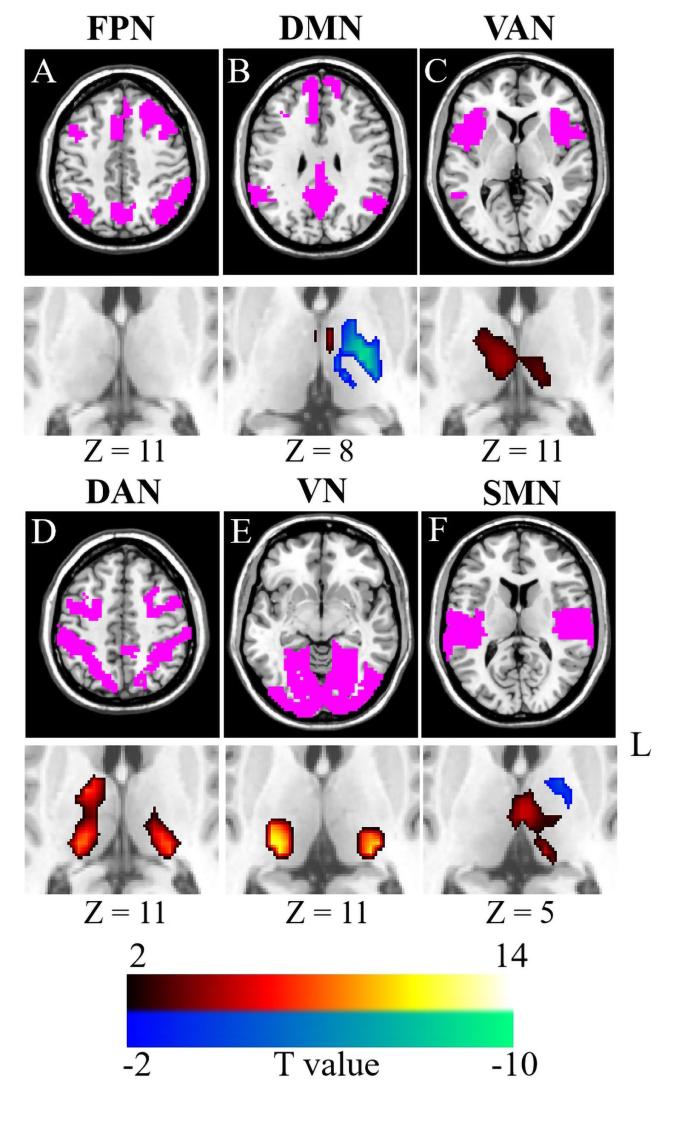


**Supplementary Figure 7.** Relationships between fluid intelligence and ISFCs between the thalamus and 7 cortical networks (A through F). Color bar signifies the T values (warm color, ISFCs increased significantly as fluid intelligence scores increased; cool color, ISFCs decreased significantly as fluid intelligence scores increased; FDR-corrected). L represents the left hemisphere. A minimum cluster size was 23 adjacent voxels. Abbreviation: FPN, frontoparietal network; DMN, default mode network; VAN, ventral attention network; DAN, dorsal attention network; VN, visual network; SMN, sensorimotor network.

1. **Split-half validation analysis**

To further verify the reliability of the results, we randomly selected half of the participants and performed the same ISFC analysis and GLM analysis. All participants were divided into seven groups with an age range of 10 years (i.e., 18-27 years old, 28-37 years old, ... 78-87 years old). The mean time courses of each brain network (17 networks in total) and time courses of thalamus (620 voxels in total) were extracted for each participant. The ISFC matrix (17× 620) was calculated for each participant within each group. GLM analyses were performed to investigate the relationship between the ISFC and age, fluid intelligence.


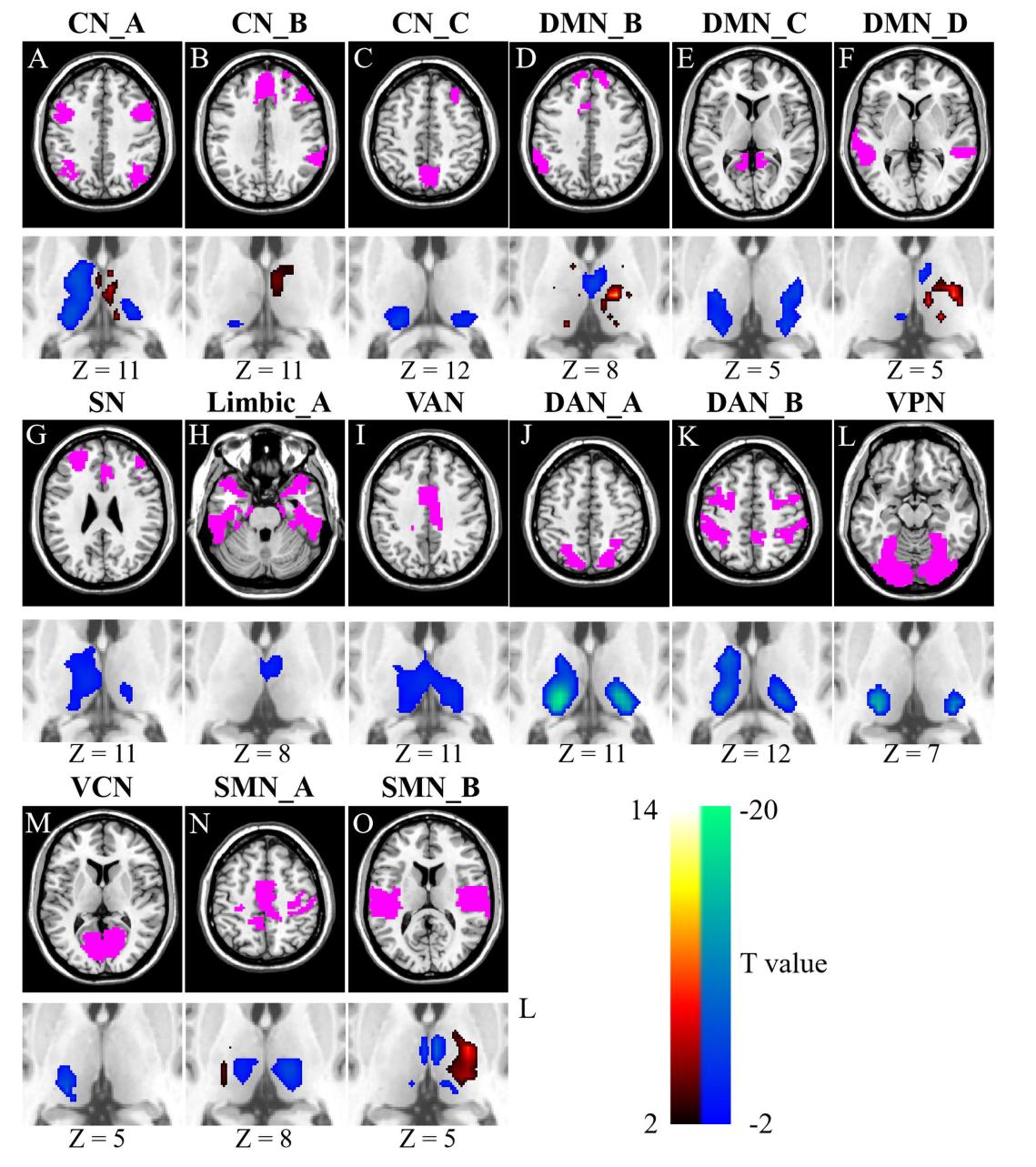


**Supplementary Figure 8.** The split-half cross validation of age-related changes of ISFCs between the thalamus and cortical networks (A through O). Color bar signifies the T values (warm color, ISFCs increased significantly as age increased; cool color, ISFCs decreased significantly as age increased; FDR-corrected). L represents the left hemisphere. A minimum cluster size was 23 adjacent voxels. Abbreviation: CN_A, control network A; CN_B, control network B; CN_C, control network C; DMN_B, default mode network B; DMN_C, default mode network C; DMN_D, default mode network D; SN, salience network; Limbic_A, limbic network A; VAN, ventral attention network; DAN_A, dorsal attention network A; DAN_B, dorsal attention network B; VPN, visual peripheral network; VCN, visual central network; SMN_A, sensorimotor network A; SMN_B, sensorimotor network B.


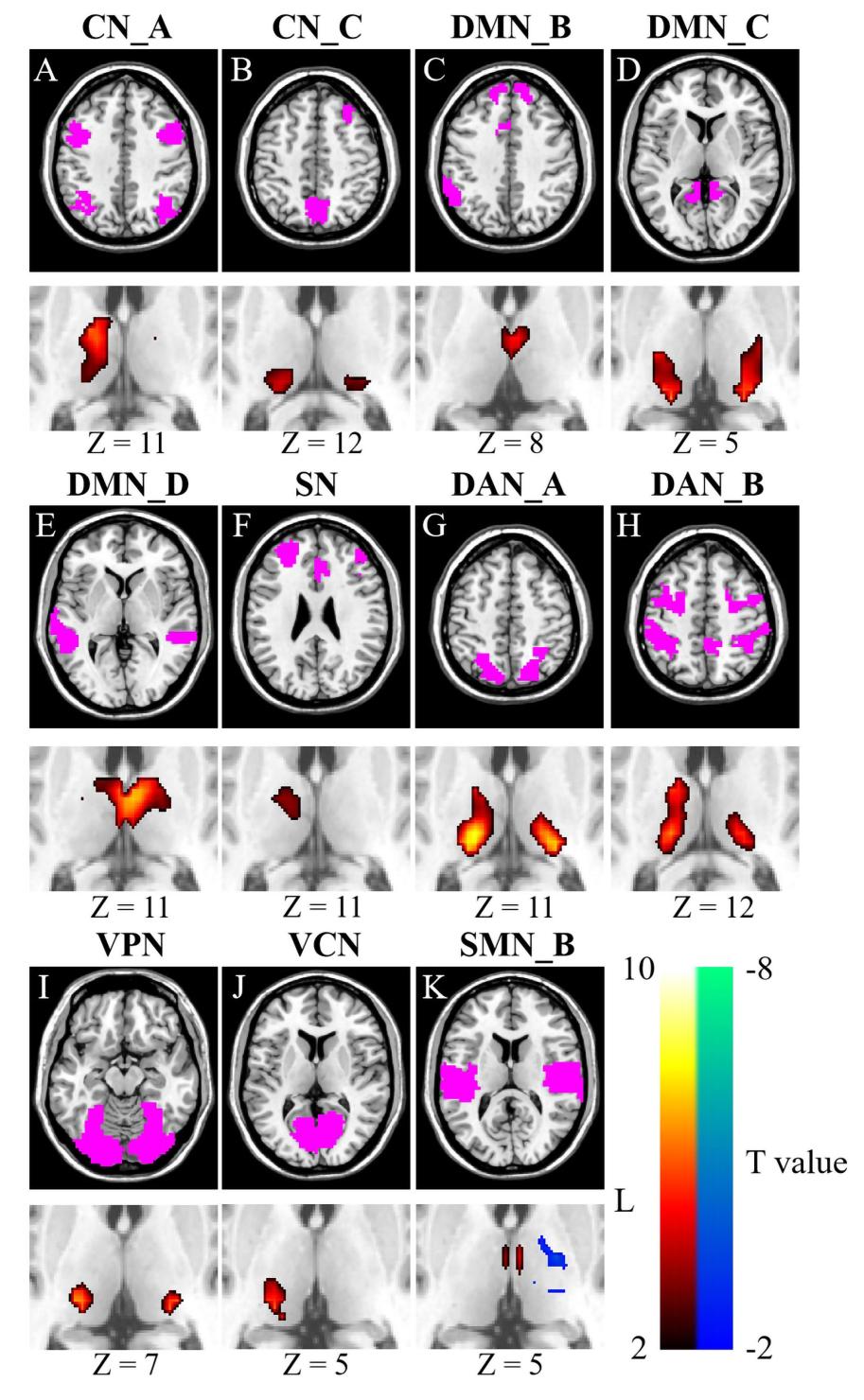


**Supplementary Figure 9.** The split-half cross validation of relationships between fluid intelligence and ISFCs between the thalamus and cortical networks (A through K). Color bar signifies the T values (warm color, ISFCs increased significantly as fluid intelligence scores increased; cool color, ISFCs decreased significantly as fluid intelligence scores increased; FDR-corrected). L represents the left hemisphere. A minimum cluster size was 23 adjacent voxels. Abbreviation: CN_A, control network A; CN_C, control network C; DMN_B, default mode network B; DMN_C, default mode network C; DMN_D, default mode network D; SN, salience network; DAN_A, dorsal attention network A; DAN_B, dorsal attention network B; VPN, visual peripheral network; VCN, visual central network; SMN_B, sensorimotor network B.
